# Identification and Evaluation of Neuropsychological Tools Used in the Assessment of Alcohol-Related Cognitive Impairment: A Systematic Review

**DOI:** 10.3389/fpsyg.2018.02618

**Published:** 2018-12-18

**Authors:** Robert Heirene, Bev John, Gareth Roderique-Davies

**Affiliations:** Addictions Research Group, University of South Wales, School of Psychology & Therapeutic Studies, Pontypridd, United Kingdom

**Keywords:** alcohol-related cognitive impairment, alcohol-related brain damage, Korsakoff's syndrome, alcohol-related dementia, neuropsychological assessment, screening, diagnosis

## Abstract

**Background:** Neuropsychological assessment is central to identifying and determining the extent of Alcohol-Related Cognitive Impairment (ARCI). The present systematic review aimed to synthesize and discuss the evidence appraising the neuropsychological tests used to assess ARCI in order to support clinicians and researchers in selecting appropriate tests for use with this population.

**Methods:** We searched for studies investigating the psychometric, diagnostic and practical values of tools used in the screening, diagnosis, and assessment of Korsakoff's Syndrome (KS), Alcohol-Related Dementia (ARD), and those with a specific diagnosis of Alcohol-Related Brain Damage (ARBD). The following databases were searched in March 2016 and again in August 2018: MEDLINE, EMBASE, Psych-INFO, ProQuest Psychology, and Science Direct. Study quality was assessed using a checklist designed by the authors to evaluate the specific factors contributing to robust and clearly reported studies in this area. A total of 43 studies were included following the screening of 3646 studies by title and abstract and 360 at full-text. Meta-analysis was not appropriate due to heterogeneity in the tests and ARCI samples investigated in the studies reviewed. Instead, review findings were narratively synthesized and divided according to five domains of assessment: cognitive screening, memory, executive function, intelligence and test batteries, and premorbid ability. Effect sizes (*d*) were calculated to supplement findings.

**Results:** Overall, several measures demonstrated sensitivity to the cognitive deficits associated with chronic alcoholism and an ability to differentiate between gradations of impairment. However, findings relating to the other psychometric qualities of the tests, including those important for the accurate assessment and monitoring of ARCI (e.g., test-retest reliability), were entirely absent or limited. Additionally, the synthesis of neuropsychological outcomes presented here supports the recent impetus for a move away from discrete diagnoses (e.g., KS, ARD) and the distinctions between them toward more broad and inclusive diagnostic conceptualizations of ARCI, thereby recognizing the heterogeneity in presentation.

**Conclusions:** Based on the evidence reviewed, provisional recommendations for appropriate tests in each domain of assessment are presented, though further validation of most tests is warranted. Review findings can support efficient and evidenced-based test-selection and guide future research in this area.

## Introduction

Chronic alcohol abuse has deleterious effects on both the function and structure of the brain (Zahr et al., 2011; Stavro et al., 2013). This is most notable in alcoholics with Wernicke-Korsakoff's Syndrome (WKS), the pathogenesis of which lies in thiamine (vitamin B1) deficiency. Alcoholics are at particular risk of developing thiamine depletion as a result of poor nutritional intake (both of the vitamin itself and others associated with its absorption [e.g., folate, vitamin B12]) and alcohol's disruptive effects on metabolism (Thomson et al., 2012). Wernicke's Encephalopathy (WE)—that is, the initial acute phase of WKS—is diagnosed on the basis of having two or more of the following symptoms: a history of nutritional deficiency, oculomotor dysfunction (nystagmus, ophthalmalgia), ataxia, and an altered mental state or memory impairment (Caine et al., 1997). If recognized early, symptoms of WE can largely be relieved via treatment with parenteral thiamine (Day et al., 2013). Yet between 56 and 85% of patients with WE will go on to develop Korsakoff's Syndrome (KS; Wood et al., 1986; Cook et al., 1998), a chronic neuropsychiatric condition characterized by profound memory dysfunction. While anterograde amnesia is pathognomonic of KS, an increasing body of evidence has indicated the cognitive deficit is not as circumscribed as once thought. For example, those with KS frequently display impaired performance on tests of Executive Function (EF) requiring planning, attention shifting, inhibition, and fluency (Brokate et al., 2003; van Oort and Kessels, 2009), and these findings are reflected in recent diagnostic criteria for the disorder (DSM-5; American Psychiatric, 2013).

It is also evident that there exists a cohort of alcoholics who present with clinically meaningful neurocognitive impairment who do not meet all the criteria for WKS or other discrete alcohol-related neurological disorders (e.g., Marchiafava–Bignami disorder). For the more impaired within this group, a diagnosis of Alcohol-Related Dementia (ARD) has been proposed (Oslin et al., 1998), although this has been met with some contention. ARD is described as a global decline in cognition with a more insidious onset than WKS, but the choice of nomenclature has been criticized as the disorder is not progressive with abstinence—unlike most dementias (Ridley et al., 2013). Debate also surrounds the etiopathogenesis of the disorder, with one line of thinking associating ARD with the direct neurotoxic effects of alcohol, including neuroinflammation and glutamate excitotoxicity occurring during withdrawal (see Ridley et al., 2013), and another suggesting ARD is simply a variation of WKS caused by thiamine depletion (Joyce, 1994). While the mechanisms of alcohol neurotoxicity are likely implicated in the neurocognitive decline of alcoholics to an extent (Sullivan and Zahr, 2008), evidence from neuropathological, neuroimaging (Harper, 2009; Zahr et al., 2011) and neuropsychological investigations (Pitel et al., 2011) is supportive of a central role of thiamine depletion. Nonetheless, it appears both processes work synergistically to generate greater damage than either in isolation (He et al., 2007).

More recently, the term Alcohol-Related Brain Damage (ARBD) has been adopted in place of other diagnostic nomenclature to describe a spectrum of neurocognitive impairment encompassing both WKS and ARD (Hayes et al., 2016). This nascent conceptualization may provide a more pragmatic nosological approach by acknowledging the heterogeneous consequences of chronic alcoholism and associated factors (e.g., nutritional deficiencies, hepatic dysfunction, cerebrovascular disorders, head injury). The most recent DSM-5 (American Psychiatric, 2013) appears more aligned with this thinking, outlining both mild and severe forms of alcohol-related neurocognitive disorder which are further divided into amnestic (i.e., KS) and non-amnestic types.

Wilson et al. (2012) have proffered criteria for ARBD as a specific diagnostic entity, adapting those provided by Oslin and Cary (2003) for ARD. They present an operational diagnostic checklist for use by hospital staff in facilitating the quick identification of those with or at risk of ARBD, with a focus both on symptomology and the frequent consequences of the disorder (e.g., regular hospital admissions). Wilson (2013) has also presented more thorough diagnostic criteria for ARBD as a broad spectrum of alcohol-related neurocognitive impairment, though neither criteria appear to have been subsequently validated. It appears that the authors encompass WKS within their definition of ARBD (based on the inclusion of individuals with WKS in their sample; Wilson et al., 2012) and thus use the term in a broad sense to capture the heterogeneous neurocognitive manifestations of alcohol abuse. However, other authors appear to use the term to describe all such manifestations that do not meet criteria for WKS (e.g., Zahr et al., 2011), in a similar way to which ARD has historically been employed. Though, as noted by the authors using the term in this manner, postulating ARBD as a distinct diagnostic entity from WKS will be subject to the same criticisms as the ARD hypothesis; namely that it may represent a variation or earlier phase of WKS (Zahr et al., 2011). Given the central role of thiamine depletion found in the cognitive decline of ostensibly “non-WKS” alcohol abusers (Pitel et al., 2011), this conclusion seems justified.

Regardless of etiological or nosological debate, the defining two features of alcohol-related neurocognitive disorders are a prolonged history of excessive alcohol use and an attributable cognitive deficit. Accordingly, neuropsychological assessment has been suggested as the most reliable method of diagnosis (Hayes et al., 2016) and been promoted for inclusion in the routine assessment of all alcoholics (Davies et al., 2005). While imaging methods are also of use in identifying alcohol-related brain changes, indices of neuronal and structural brain change derived from such measures provide limited information about the functional implications of this damage, which are likely of greater clinical interest. In contrast, neuropsychological testing can characterize both the type and extent of cognitive deficit (thereby guiding rehabilitation efforts), inform assessments of capacity, and be used to monitor improvements over time (Hayes et al., 2016).

Multiple studies have explored the psychometric and practical value of various neuropsychological tools for the assessment of Alcohol-Related Cognitive Impairment (ARCI), providing a wealth of information regarding the utility of various tests (e.g., Wester et al., 2013b; Rensen et al., 2015). Systematic reviews and meta-analyses of such studies in other populations (e.g., dementia) are common (e.g., Tsoi et al., 2015; Carson et al., 2018), though there remains a lacuna in the area of ARCI assessment. A review was recently published by Horton et al. (2015a) which discussed neuropsychological testing and other methods of assessment (psychological, nutritional etc.) used with this group. However, the review touched only superficially on the complexities of ARCI assessment, failed to include multiple relevant studies (e.g., Kopelman, 1991; Taylor and Heaton, 2001), and omitted investigations of ARD and ARBD participants.

The absence of a rigorous systematic review complicates evidence-based test selection for those involved in ARCI assessment, making it a time consuming and complex process of comparison across multiple tests and studies. At present, the literature surrounding test utility is less-established for ARCI than disorders such as Alzheimer's Disease, warranting a more broad and comprehensive review of the existing evidence-base in order to discuss its current status and guide future research directions. Accordingly, the aims of the systematic review presented here were to: [1] identify the neuropsychological tools most commonly used in the screening, diagnosis, and assessment of ARCI, and [2] synthesize and discuss all findings relating to the diagnostic, psychometric and practical merits of these tests within the assessment of this population. We adopted a broad view of ARCI as a conceptual category of disorders, including KS, ARD, and ARBD as a specific diagnostic entity. While, as suggested above, all cases of ARCI may be variants of the WKS condition, we searched for studies assessing individuals with diagnoses of ARD and ARBD based on the use of these distinctions within the existing literature. It was recognized that ARD and ARBD may refer to the same purported condition and that the particular term used could reflect the year of publication, as opposed to differences in the symptomology of those diagnosed with ARBD over ARD, or vice versa. The inclusion of studies assessing each of these three groups also allowed for further evaluation of the validity of these diagnostic distinctions from a neuropsychological perspective. Studies assessing WE were omitted due to a greater focus on the assessment of physical symptoms (i.e., Caine et al., 1997 criteria), thiamine blood levels, and neuroimaging outcomes (Lough, 2012) when making diagnostic decisions.

## Methods

A review protocol was developed and registered with PROSPERO in December 2015 (Registration No. CRD42015030209) and later published (Heirene et al., 2016). We provide a brief overview of the review methods here (including any deviations from our protocol), though recommend referring to our protocol for further details. In developing the protocol and conducting the review we consulted the PRISMA-P (Moher et al., 2015; Shamseer et al., 2015) and PRISMA guidelines (Liberati et al., 2009) and additional guidance on conducting systematic reviews in neuropsychology (Gates and March, 2016) and narrative synthesis (Popay et al., 2006; Centre for Reviews Dissemination, 2009).

### Data Sources and Study Selection

We searched for studies using standardized, normatively defined neuropsychological tools in the screening, diagnosis or assessment of KS, ARD, and ARBD. Studies assessing alcohol-dependent persons without one of the above ARCI diagnoses were omitted as the focus of the review was on the ability of neuropsychological tests to identify and assess those with clinically significant cognitive impairment (i.e., meeting diagnostic criteria) and to differentiate these individuals from those with little or no impairment. We made two deviations from the initial aim of including only standardized, normatively defined neuropsychological tests. The first to include studies employing standardized observational measures of confabulation which are not normatively defined, enabling a more comprehensive review of ARCI assessment tools. The second to include a study evaluating the utility of the Brown-Peterson paradigm (a non-standardized test with normative data available) to allow a more detailed discussion of working memory assessment. We also deviated from our protocol by using the term ARCI over ARBD to frame this review as the former is more widely used outside of the UK.

**Figure 1:**
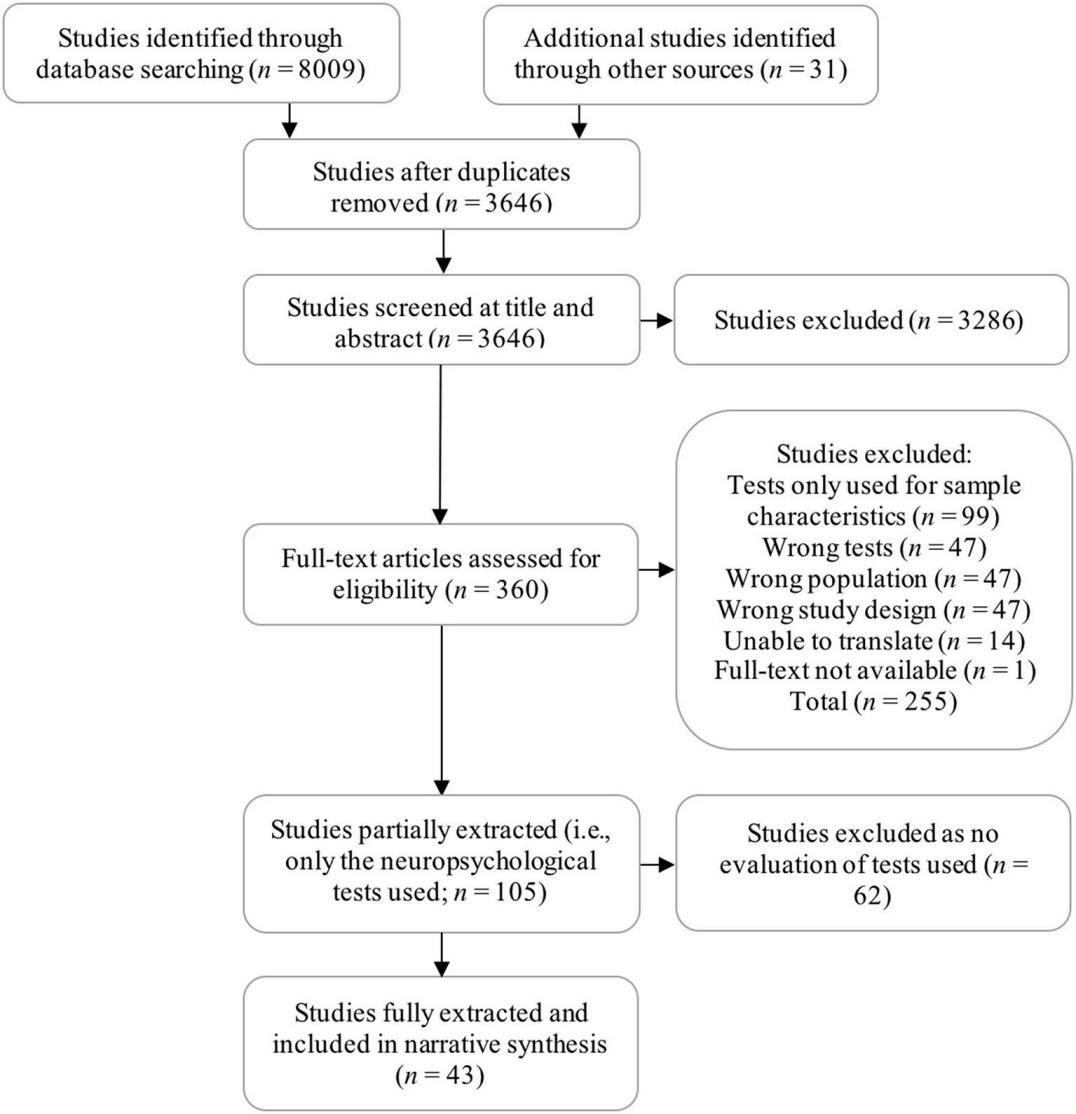
Study selection process: PRISMA Flow-Chart.

The following databases were searched on January 19th 2016 and again in August 2018: MEDLINE, EMBASE, Psych-INFO, ProQuest Psychology, and Science Direct. To achieve both aims of the review the process of study selection and subsequent data extraction was divided into two consecutive phases. In the first phase, we identified 105 studies meeting our original eligibility criteria (i.e., standardized test used to assess ARCI). From these, we extracted only the type of ARCI sample assessed and the tool used, addressing aim 1 (i.e., identify the tools used). In phase two, 43 studies were identified from within this selection which provided some evaluation of the psychometric, diagnostic or practical merits of the tools used. These studies were extracted in full using a table designed by the authors and presented in our protocol (Heirene et al., 2016). Extracted data from phase two was used to produce a narrative synthesis of findings focused on addressing aim 2 (i.e., evaluation of the measures) (see Figure 1 for an overview of the study selection process).

Title and abstract and full-text screening were conducted independently by two authors (RH & GR-D) via Covidence online systematic review software. A pilot full-text screen of 20 texts was conducted to check inter-reviewer consistency. A Kappa statistic of .839 (*P* <.001) was achieved from this process, indicating excellent consistency between reviewers (Viera and Garrett, 2005). Phase two of extraction and study appraisal was also completed independently by two authors (RH & BJ), though phase one extraction was carried out by one author (RH).

### Quality Assessment

All studies extracted in full were appraised using the quality assessment checklist designed by the authors and presented in our protocol (Heirene et al., 2016). We made minor updates to this checklist to provide a less ambiguous and more comprehensive assessment of study rigor (see Supplemental Document 1). We discuss the outcomes of the study appraisal at the end of the findings section, though we also present multiple indices of study quality in the tables of findings (e.g., ensuring a sufficient period of abstinence achieved prior to testing) and highlight any notable methodological weaknesses or strengths of studies when discussing their contribution to the review.

### Synthesis of Findings

As anticipated in our protocol, meta-analysis was not appropriate for the synthesis of findings due to heterogeneity in the tests used, the samples of focus, comparator groups, and the outcomes reported. Instead, findings are synthesized in the form of a narrative review, with tables used throughout to present key outcomes. Findings are divided into five sections covering cognitive screening instruments, memory, EF, intelligence and test batteries, and premorbid intelligence. Where relevant and possible, effect sizes were calculated for between-group comparisons (e.g., ARCI vs. controls) to supplement review findings (Sullivan and Feinn, 2012). Cohen's *d* was selected as a commonly used effect size which calculates the standardized difference in means between groups and can facilitate the relative comparisons of each test's sensitivity (Larner, 2014). Based on Cohen's (1992) original suggestion, effects are classified as small (*d* = 0.2), medium (*d* = 0.5), or large (*d* ≥ 0.8). However, these cut-off points are somewhat arbitrary. All effect sizes should be considered in conjunction with the methodological quality of the study (e.g., sample size, abstinent participants) before reaching conclusions regarding the size of the effect or the implications of this for the test used. Sensitivity and specificity values for tests were also calculated where relevant; though few studies provided the requisite information (all reviewer calculated outcomes are presented inside squared brackets [] in tables).

## Findings

### Study Characteristics

The 105 studies included in phase one of data extraction ranged in year of publication from 1968 to 2017. Ninety studies assessed KS participants, 15 ARD, six ARBD, and four included samples described as having mild alcohol-related impairment not fitting KS or ARD criteria (ALC), one of which was diagnosed according to the DSM-5's alcohol-related Mild Neurocognitive Disorder. The most commonly used tests are presented in Table 1 according to each domain of cognitive assessment discussed in the narrative synthesis (for a complete list of all neuropsychological tests used within the reviewed studies see Supplemental Document 2).

**Table 1:** Standardized neuropsychological tools used in the assessment of ARCI.

**Cognitive screening**	**Memory**	**Executive function**	**Intelligence and test batteries**	**Premorbid function**
Mini-Mental Status Examination (*n* = 23)^#^	Wechsler Memory Scale-I/II/III (*n* = 38/12/4)^#^	FAS Verbal Fluency (*n* = 20)^#^	Wechsler Adult Intelligence Scale-I/II/III (*n* = 41/30/11)^#^	National Adult Reading Test/-R (*n* = 41/2)^#^
Dementia Rating Scale (*n* = 6)^#^	Rey-Osterrieth Complex Figure Test (*n* = 21)	Wisconsin/Modified Card Sorting Test (*n* = 14/10)^#^	Raven's Progressive Matrices (*n* = 4)	Mehrfacjwajhi-Wortschaz Test (German; *n* = 3)
CAMCOG (*n* = 6)^#^	Rivermead Behavioral Memory Test/-3 (*n* = 6/5)^#^	Stroop Word-Color Test (*n* = 17)	Consortium to Establish a Registry for Alzheimer's Disease (*n* = 4)	Wechsler Adult Intelligence Scale - Vocabulary Test (*n* = 2)^#^
Montreal Cognitive Assessment (*n* = 2)^#^	California Verbal Learning Test (*n* = 10)^#^	Trial Making Test (*n* = 13)	Leistungs-prüf-system (German; *n* = 4)	Wechsler Test of Adult Reading (*n* = 1)
Addenbrook's Cognitive Examination (*n* = 1)	Rey-Osterrieth Auditory Verbal Learning Test (*n* = 10)	Cognitive Estimation Test/-Shortened (*n* = 4/2)^#^	Halstead-Reitan Battery (*n* = 3)	

*Top five most used tests in each domain of assessment presented. For a complete summary of all tests used along with tests authors and associated references see Supplemental Document 2*;

# *Test evaluated by studies within the narrative synthesis*.

The final 43 studies included in phase two of data extraction spanned a range of 40-years from 1977 to 2017. Consonant with the studies included in the first phase of extraction, the majority focused on KS participants (*n* = 39), with few investigating ARD (*n* = 4), ALC (*n* = 4) or those with a diagnosis of ARBD (*n* = 2). Adding to the confusion surrounding the use of ARBD as a specific diagnosis that was highlighted in the introduction, neither of the two studies which assessed participants with this diagnosis (i.e., Welch et al., 1997; Horton et al., 2015b) presented criteria for the condition or clearly described the definition of ARBD which they adopted. However, it appears that Welch et al. (1997) used the term to refer to milder neurocognitive impairment not meeting criteria for WKS, while Horton et al. (2015b) used the term more broadly; though it is unclear whether this included individuals with WKS diagnoses.

Most studies employed a between groups design (*n* = 36), though four assessed only a single group, two assessed multiple clinical groups individually, and one was a case-study. Various outcomes of relevance to the review were reported, with the most common relating to the diagnostic value of tests, including sensitivity and specificity values (*n* = 23); the use of tests for discriminating between groups (*n* = 13); positive and negative predictive values (*n* = 1); and likelihood ratios (*n* = 1). Twelve studies commented on the practical values of tests, including ease or time of administration or the availability of parallel versions. Outcomes relating to validity were common, including convergent (*n* = 11), construct (*n* = 11), predictive (*n* = 2), concurrent (*n* = 1), and content validity (*n* = 1); while only four studies reported reliability values (inter-rater = 2; test-retest = 1; internal = 1).

### Cognitive Screening Instruments

Five reviewed studies evaluated Cognitive Screening Instruments (CSIs) in those with ARCI (Table 2 presents a summary of test and study details along with key outcomes). The Montreal Cognitive Assessment (MoCA; Nasreddine et al., 2005) was assessed by two studies and has received the comparatively greatest psychometric evaluation. In Wester et al. (2013c), the MoCA effectively differentiated between KS and ALC groups, between KS and controls, and between ALC and controls, mostly all with good to excellent sensitivity and specificity levels provided the cut-off score was adjusted accordingly. Only the KS vs. ALC MoCA comparison did not reach optimal 80% sensitivity and 60% specificity criteria cited by the authors (Blake et al., 2002).

**Table 2:** Cognitive screening instruments.

**Test**	**Summary**	**Functions assessed**	**Study**	**ARCI samples**	**Comparators**	**Outcomes**					
						**Between group comparisons**	**Sensitivity (sens) & specificity (spec)**	**Validity**	**Positive & negative predictive values**	**Positive & negative likelihood ratios**	**Practical considerations**
CAM-COG	The cognitive section of the Cambridge Examination for Mental disorders. Contains 67 items (including all those of the MMSE) scored out of 107 with ≤ 80 used as a cut-off score for dementia. Administration time: 25 min.	Orientation Language Memory Learning Attention Praxis Calculation Abstract thinking Perception.	Deary et al., 1991	KS (*n* = 11)_r,a_	Alzheimer's Dementia (AD; *n* = 10)_i_ CG (*n* = 11)i	Total score: AD < KS^*^	NA	Convergent:Culture Fair Intelligence Test: *r* = 0.68.WAIS-Digit Symbol Test: *r* = 0.83.Digit Symbol correlated with 7 (of) 11 CAMCOG subtests.	NA	NA	NA
			Woodburn and Johnstone, 1999a,b	ARD (*n* = 13; DSM-III criteria for dementia)_r_	Alzheimer's Disease (*n* = 60) Multi-infarct (*n* = 13) & mixed (*n* = 24) dementia	ARD> than at least 1 other dementia group^*^-^***^ on all variables.	Narrative: authors stated ARD can be distinguished from other dementias using the CAMCOG based on an overall milder pattern of impairment & marginally improved performance on repeat testing.	NA	NA	NA	Narrative: easily administered & covers a range of dementia severities & etiologies.
MMSE	A brief cognitive assessment designed for dementia screening. Provides sub-test & overall scores with a maximum of 30 & standard cut-off point of <24. Administration time: 5–10 min.	Orientation Attention Calculation Language Memory (verbal): immediate & delayed recall Comprehension Copying.	Deary et al., 1991	KS (*n* = 11)_r,a_	Alzheimer's Dementia (AD; *n* = 10)_i_ CG (*n* = 11)_i_	AD < KS^*^ [*d = –*1.1708].	NA	Convergent: Cattel Culture Fair Intelligence Test: *r* = 0.70. WAIS-Digit Symbol Test: *r* = 0.69.	NA	NA	NA
			Oudman et al., 2014	KS (*n* = 30; DSM-IV-TR, Kopelman, 2002)_r,a_	CG (*n* = 30)_d,i_	KS < CG^***^ [*d* = 1.881].	Standard cut-off score (<24): ROC AUC 0 = 0.92 (95% CI = 0.85–0.98, SE = 0.033), sens = 73.3%, spec = 93.3%, participants correctly classified = 78.3%. Optimal cut-off (<26/27): sens = 90/100%, spec = 83.3/73.3%, participants correctly classified = 81.7/85%.	Convergent (KS): MMSE & MoCA total score: *r* = 0.73^**^.	Standard cut-off score (<24): PPV = 90.4%, NPV = 71.8%. Optimal cut-off (<26/27): PPV = 82.8/77.1%, NPV = 80.6/88%.	Standard cut-off score (<24): PLR = 10.9, NLR = 0.3. Optimal cut-off (<26/27): PLR = 5.4/3.8, NLR = 0.1/0.	NA
MoCA	Brief cognitive screening measure designed for the detection of mild cognitive impairment & dementia. Provides sub-test & overall scores with a maximum of 30 and standard cut-off of <26. Administration time: 10 min.	Memory (verbal): immediate, delayed & (if required) cued recall Executive function (EF) Attention & concentration Language Visuospatial abilities Orientation.	Wester et al., 2013c	KS (*n* = 20; DSM-IV-TR)_r_	ALC (*n* = 26)_d,i_ CG (*n* = 33)_i_	Total score: KS < CG^***^ [*d* = 2.57], KS < ALC^**^ [*d* = 0.85]. Memory: KS < CG-ALC^***^ [KS vs. CG: *d* = 3.23; KS vs. ALC: *d* = 1.43], Orientation: KS < CG-ALC^***^ [KS vs. CG: *d* = 1.96; KS vs. ALC: *d* = 1.51], EF: KS-ALC < CG^*^ [KS vs. CG: *d* = 0.83; KS vs. ALC: *d* = 0.11],	KS vs. CG (cut-off score of ≤ 23): ROC AUC = 0.96***, sens: 88%, spec: 95%. KS vs. ALC (cut-off ≤ 20): ROC AUC = 0.75**, sens: 73%, spec: 75%. ALC vs. CG (cut-off ≤ 24): ROC AUC = 0.82***, sens: 85%, spec: 69%. Severe memory impairment group vs. no impairment^#^	NA	NA	NA	NA
						remaining subtests: KS-ALC-CG^ns^.	(cut-off ≤ 23): ROC AUC = 0.96***, sens = 91%, spec = 88%. Severe memory impairment group vs. mild impairment^#^ (cut-off ≤ 20): ROC AUC = 0.75**, sens = 81%, spec = 69%. Mild memory impairment group vs. no impairment^#^ (cut-off ≤ 24): ROC AUC = 0.82***, sens = 88%, spec = 71%.				
			Oudman et al., 2014	KS (*n* = 30; DSM-IV-TR, Kopelman, 2002)_r,a_	CG (*n* = 30)_d,i_	Total score: KS < CG^***^ [*d* = 2.93].	Standard cut-off score (<26): ROC AUC = 1 (95% CI = 0-1, SE = 0.003), sens = 100%, spec = 63.3%, participants correctly classified = 81.7%. Optimal cut-off (<22/23): sens = 100/100%, spec = 100/96.7%, participants correctly classified = 95/98.3%.	NA	Standard cut-off score (<26): PPV = 73.2%, NPV = 100%. Optimal cut-off (<22/23): PPV = 100/96.8%, NPV = 90.9/100%.	Standard cut-off score (<26): PLR = 2.7, NLR = 0. Optimal cut-off (<22/23): PLR = infinite/30.3, NLR = 0/0.	NA

*Tests: CAMCOG, Cambridge Cognitive Examination; MMSE, Mini-Mental Status Examination; MoCA, Montreal Cognitive Assessment; Wechsler Adult Intelligence Scale*.

*Samples: AL, Alcoholic group (no diagnosis of cognitive impairment); ALC, Alcoholic group with evidence of cognitive impairment not fitting KS or ARD criteria; ARD, Alcohol-Related Dementia; CG, Control Group; KS, Korsakoff's Syndrome*.

*Study quality: _r_, reference standard used to confirm diagnosis; _a_, abstinent for 6 weeks prior to testing; _d_, demographics: comparator group matched for age and gender with ARCI sample or differences accounted for in analyses; _i_, intelligence: comparator group matched for premorbid IQ and or education or differences accounted for in analyses*.

*Outcomes: NA, not assessed*;

*, **, *** *significant at the 0.05, 0.01 and 0.001 alpha level respectively*;

ns *, not significant; d, Cohen's d effect size (minus values indicate the ARCI group performed better than comparator); ROC, Receiver Operating Characteristics; AUC, Area Under Curve; CI, Confidence Interval; PPV, Positive Predictive Value; NPV, Negative Predictive Value; PLR, Positive Likelihood Ratio; NLR, Negative Likelihood Ratio*;

# *, divided according to RBMT-3 General Memory Index score*.

Further support for the MoCA has come from Oudman et al. (2014), who directly compared the psychometric properties of the MoCA and the Mini-Mental State Examination (MMSE; Folstein et al., 1975) in a KS and control sample. Overall, both the MoCA and MMSE demonstrated good to excellent sensitivity and specificity, with the MoCA the superior of the two. Reviewer calculated effect sizes support this finding, indicating that the MoCA produced substantially larger relative differences when comparing controls and KS than the MMSE. Additionally, a MoCA cut-off score of <23 produced nearly perfect positive and negative predictive values and likelihood ratios in Oudman et al. (2014). However, while these values support the screening capabilities of the tool, they should be interpreted with caution as the equal proportion of KS to controls investigated by Oudman and colleagues is unreflective of typical clinical environments, and thus the values would likely decrease in such settings. As in Wester et al. (2013c), adjustments to the cut-off score of both CSIs were required by Oudman et al. to result in the best possible discriminatory abilities. Poor sensitivity of the MMSE to KS warranted a rise in the standard cut-off score of <24 to between 25 and 27 to produce optimal sensitivity and specificity levels, with <26/27 being most able to discriminate between KS and controls (Wester et al., 2013c). For the MoCA, the combined findings of Oudman et al. and Wester et al. suggest a cut-off score of ≤ 23 is needed to distinguish between unimpaired individuals and KS, ≤ 24 for unimpaired vs. ALC, and ≤ 20 for ALC vs. KS. Nonetheless, caution should be exercised when reducing or increasing cut-off scores to be conscious of increased false negatives and false positives, respectively.

Although Oudman et al.'s (2014) findings suggest the MMSE may have value in screening for ARCI, the test's utility is restricted by the absence of items specifically indexing executive abilities. Additionally, the memory component of the MMSE has been criticized for its simplicity and lack of sensitivity to alcohol-related memory disorders (Squire and Shimamura, 1986; Kopelman et al., 2009). The cognitive assessment component of the CAMDEX (Roth et al., 1986), the CAMCOG, includes all items from the MMSE in addition to more difficult tests of memory and measures of EF, and thus may be more suitable for ARCI screening. However, Deary et al. (1991) found several of the CAMCOG subtests (7 of 11) correlated strongly with the Digit Symbol Substitution test of visuospatial processing, which they stated indicates high redundancy within much of the CAMCOG. Still, research evaluating the CAMCOG in this population is scant–and nonexistent for the updated CAMCOG-R (Roth et al., 1999).

### Memory

In total, 22 studies evaluated tests of memory, which are further divided into tests of episodic, autobiographical, procedural, and working memory and confabulation.

#### Episodic Memory

Seventeen studies evaluated neuropsychological tools designed to assess episodic memory function (see Table 3). Two tests frequently employed were the Rivermead Behavioral Memory Test (RBMT; Wilson et al., 1989) and the California Verbal Learning Test (CVLT; Delis et al., 1987). The RBMT was designed as an ecologically focused memory test, offering clinically and practically useful information about a person's memory deficit. The RBMT includes various tasks one might encounter in regular life, such as having to remember an appointment or pass on a message; though, to date, no studies have assessed the RBMT's ability to predict day-to-day memory function in those with ARCI.

**Table 3:** Tests of episodic memory.

**Test**	**Summary**	**Aspects of memory assessed**	**Study**	**ARCI samples**	**Comparators**	**Outcomes**				
						**Between group comparisons**	**Sensitivity (sens) & specificity (spec)**	**Validity**	**Reliability**	**Practical considerations**
3W3S	A memory screening tool designed for bedside assessment. Requires the copying & subsequent retrieval of 3 words & 3 shapes immediately, then after 5, 15, and 30 min. Administration time: ~40 min.	Verbal & visual memory Incidental, & delayed recall Learning Recognition memory.	Weintraub et al., 2000	KS (*n* = 7)	Probable Alzheimer's Disease (PRAD; *n* = 21) CG (*n* = 14)	Copy (words/shapes): KS-CG^ns^, Learning trials to criterion: KS < CG^***^ [*d* = 1.54], Incidental recall (words/shapes): KS < CG^*^ [*d* = 0.63/1.17], Acquisition: KS-CG^ns^, Delay recall (words/shapes): KS < CG^***^ [*d* = 2.69/2.29], Recognition accuracy (words/shapes): KS < CG^***^ [*d* = 1.19/0.83]. All variables: KS-PRAD^ns^.	NA	Convergent (controls): 3W3S & delayed recall of the CERAD word list: *r* = 0.84^**^ (words), *r* = 0.73^*^ (shapes). Narrative (content): assesses various aspects of memory not covered by other screening tools.	NA	Narrative: the test is easily given in various settings with little equipment & can be adjusted for participants with higher abilities (i.e., increasing stimuli) or with non-English speakers (i.e., language-specific words).
CVLT	Verbal memory test containing 2, 16-word lists. List A: 4 words in 4 semantic categories (e.g., animals). List B: an interference list containing 2 new semantic categories & 2 from List A. The first 5 trials consist of immediate recall of words from List A, then List B is presented for one trial, followed by short-delay free & cued-recall trials of List A & again after 20 min (long-delay). Finally, after 10 min an optional forced choice yes/no recognition trial is given. Hand scoring possible, though software provides greater depth of analysis & is less timely. Administration time: 20 min for standard version, 50 min with the addition of the 10- & 20-min delays.	Cued & free recall Recognition Immediate & delayed recall Various indices of learning & error (e.g., total intrusions, semantically related & unrelated intrusions, pro- & retro-active interference etc.).	Delis et al., 1991	KS (*n* = 8; Butters and Cermak's, 1980; criteria for KS)	Alzheimer's Dementia (AD; *n* = 20)_d_ Huntington's Disease (HD; *n* = 20)_d_	All 20 CVLT variables: KS-AD^ns^. HD CVLT profile qualitatively different: better recognition performance & less intrusions.	Narrative: the CVLT can distinguish HD from AD, but not between KS & AD, where repeat testing is required to identify (if KS) the absence of deterioration.	NA	NA	NA
			Brokate et al., 2003	KS (*n* = 25; ICD-10; Caine et al., 1997)_r_	AL (*n* = 23)_i_ CG (*n* = 21)_i_	Sig. effect of group on all variables**. [Trials 1-5: KS vs. AL/CG: *d* = 3.02/2.64], [Trial 5: KS vs. AL/CG: *d* = 2.94/2.53], [Free Recall: KS vs. AL/CG: *d* = 2.41/2.10], [Cued Recall: 1-5, KS vs. AL/CG: *d* = 1.71/2.02], [Perseverations: KS vs. AL/CG: *d* = 1.19/0.34], [Discriminability: KS vs. AL/CG: *d* = 1.04/1.11].	Dichotomized learning score ranges between KS (3-9 words) & AL (10-16 words) groups. Narrative: 5^th^ learning trial showed the highest degree of selectivity.	NA	NA	NA
			Wester et al., 2014	KS (*n* = 136; DSM-IV-TR; Kopelman, 2002)_r,a_	ALC (*n* = 73)_d,i_ AL (*n* = 24)_d,i_	All variables: KS < ALC^***^ [*d*s range = 0.77 (Rate of forgetting)−1.99 (ST Free Recall); Sig differences between ALC & AL for ST Free-Recall^**^ [*d* = 2.16], LT Free Recall^*^ [*d* = 2.24], & Rate of forgetting^*^ [*d* = 1.50].	CVLT indices contributed more to group prediction in discrimination analysis than RBMT indices, with free & cued recall variables better than recognition.	NA	NA	NA
			Rensen et al., 2017	KS (*n* = 51; DSM-5; Kopelman et al., 2009)_r_	–	NA	98.1% of sample more than 1.5 *SD* from the mean for overall performance on Trials 1-5. 94.2% showed rapid forgetting in delayed testing compared with norm data.	NA	NA	NA
DPT	Long-term memory test requiring participants to freely recall & distinguish between previously seen stimuli (visual & verbal). Administration time: 35–40 min.	Visual & verbal free-recall & recognition memory Immediate & delayed memory.	Maharasingam et al., 2013	KS (*n* = 15; Caine et al., 1997)_r_	AL (*n* = 16)_d,i_	KS < AL***, η^2^ = 0.42 [*d* = 1.66].	Total score (cut-off: 1.5 *SD* below mean): all KS & 10 ALs classed as impaired [sens = 100%, spec = 37.5%].	NA	NA	NA
FCSRT	Participant shown 16 pictures on 4 cards & asked to recall freely & (if necessary) with a category cue (e.g., fruit) the pictures after a 20-s of counting backwards. Includes an initial phase where all pictures shown & recalled to ensure encoding has occurred & 3 learning trials. Administration time: ~50 min	Visual memory Cued & free recall Short & long-delay recall. Learning.	Pitel et al., 2008	KS (*n* = 14; DSM-IV)_r_	AL (*n* = 40)_d,i_ CG (*n* = 55)_d,i_	Total Free-Recall: KS < AL^***^ [*d* = 3.54], KS < CG^***^ [*d* = 5.93], AL < CG^*^ [*d* = 0.84].	Dichotomized score ranges between KS & AL groups on the total free-recall score. Narrative: authors stated the FCSRT can be used effectively to differentiate KS & AL.	NA		NA
RBMT	RBMT-I: Ecologically focused battery assessment consisting of 9 sub-tests, 3 of which can be further separated into immediate & delayed recall trials. Provides sub-test scores representing various memory functions *(M =* 10, S*D* = 3), a Screening Score & Standardized Profile Score. Administration time: 25 min.	Episodic, verbal, non-verbal, spatial & prospective memory Immediate & delayed recall Orientation.	Duffy and O'Carroll, 1994	KS (*n* = 18; DSM-III-TR)_r,a_	Schizophrenia (SP; *n* = 40)_d,i_	Standardized Profile Score: KS < SP*^**^.	Screening Score: 100% of KS & 5% of SP in the severely impaired range (0-2, out of 12).	NA	NA	Narrative: test is relatively undemanding & clinically orientated.
			Wester, 2007	KS (*n* = 322; DSM-IV)^r^	English Normative sample (EN) Dutch normative sample engaged in memory training course (DN)	NA	Screening Score (KS/EN, DN): Severely impaired: 53.4/0/2.4%; Moderately impaired: 95.3/0/9.6%; Mildly impaired: 100/17.1/64%. Standard Profile Score (KS/EN, DN): Severely impaired: 63.4/0/2.4%; Moderately impaired: 96.9/0.9/8%; Mildly impaired: 100/31.6/58.4%.	NA	NA	[All 322 KS able to complete the test in full, indicating good tolerance of the test in KS].
			Wester et al., 2014	KS (*n* = 136; DSM-IV-TR; Kopelman, 2002)_r,a_ ALC (*n* = 73)	ALC (*n* = 73)_d,i_ AL (*n* = 24)_d,i_	KS < ALC-CG^***^ on all subtests excluding Belongings^**^ [KS vs. ALC: *d*s range = 0.44 (Belongings) – 1.26 (Story Delayed); KS vs. AL: *d*s range = 0.71 (Face Recog) – 1.48 (First Name)], [Total Profile Score: KS vs. ALC/AL: *d* = 1.67/2.09], [Screening Score, KS vs. ALC/AL: *d* = 1.65/1.95].	Immediate & delayed testing of the Story Recall subtest contributed more than any other subtest to a between groups discriminant analysis.	NA	NA	NA
	RMBT-3: Modified version with the addition of more stimuli & trials for some subtests, the inclusion of the “Novel subtest” (immediate & delayed) & a larger normative data sample. Includes a General Memory Index (GMI) with a mean of 100 & *SD* of 15. Administration time: 30 min.	As for the RBMT-I with the addition of procedural learning.	Wester et al., 2013b	KS (*n* = 49; DSM-IV-TR; Kopelman, 2002)_r,a_	ALC (*n* = 49)_d,i_ CG (*n* = 53)_i_	KS < CG^***^ on all subtests with large effect sizes reported [*d*s range = 0.92 (Story Recall Immed) – 4.03 (Pictures)], KS < ALC^***^ on all subtests aside from Story Recall-Immed^ns^ [*d*s range = 0.45 (Story Recall-Immed) – 1.33 (Novel Task Delayed)].	GMI: KS vs. ALC groups (cut-off <67.5) ROC: AUC = 0.85, 95% CI: 0.78-0.93***; ALC vs. CG (cut-off <87.5) ROC: AUC = 0.083, 95% CI 0.75–0.91*^**^.	NA	NA	NA
			Wester et al., 2013a	Combined KS (*n* = 15; DSM-IV-TR; Kopelman, 2002) & ALC (*n* = 10) (ARCI group)_r,a_	CG (*n* = 25)_d,i_	ARCI < CG^***^ on Total Score [RBMT-3: *d* = 3.18; RBMT-I: *d* = 1.89] & 10 (of 14) subtests [*d*s range = 1.2 (Names) – 3.92 (Route Delayed)]. ARCI-CG^ns^ on Belongings, Appointments, Pictures, & Messages-Immed [*d*s range = 0.83 (Appointment) – 1.08 (Messages Immed)].	Fewer of the CG classed as impaired on RMBT-3 subtests than RBMT-I subtests. [Effect sizes (*d*) for ARCI vs. CG comparisons were larger for the RBMT-3 than the RBMT-1 on 8 (of 12) variables (not including the RBMT-3's Novel Task variables); RBMT-I effect sizes larger for: Names, Belongings, Appointments, & Pictures].	Produced less ceiling & floor-effects than the RBMT-I.	NA	Narrative: multiple parallel versions available & translations in multiple languages.
WMS	The WMS-I memory assessment contained 7 subtests: Personal & Current Information, Orientation, Mental Control, Logical Memory, Memory Span, Visual Reproduction, & Associate Learning. The first 3 of these were designed to screen for problems with long-term memory, disorientation & attention & for aphasia. All 7 tests contributed to a Memory Quotient (MQ) score with a mean of 100 & *SD* of 15, allowing direct comparison with WAIS intelligence scores.	Long-term semantic memory Orientation Immediate recall: visual & verbal Working memory: visual & auditory.	Kopelman, 1986	KS (*n* = 16; Cutting, 1978)_a_	CG (*n* = 16)_i_ Depression (*n* = 16)_i_ Alzheimer's Dementia (AD; *n* = 16)_i_	Logical Memory (Immediate, Delayed, & Retention [delay ÷ immediate]) subtest: KS < Depression***, KS-AD^ns^.	[Sens & spec for KS vs. CG) = 100% for following variables: Logical Memory Immediate (cut-off <7), Logical Memory Delayed (cut-off <5), Logical Memory Retention = delay ÷ immediate (cut-off <50% retention)].	Narrative: KS & AD can be discriminated based on tests of small quantities of verbal information, such as digit spans (AD < KS), but not on other WMS tests.	NA	NA
			Alekoumbides et al., 1987	KS (*n* = 15)	CG (*n* = 118; contained unclear no. with neurotic disorders or psychosis) psychosis)	Logical Memory, Visual Reproduction & Associate Learning: KS < CG**, Digits Forward & Backward: KS-CG^ns^.	Narrative: authors concluded depressed Logical Memory, Visual Reproduction, & Associate Learning test scores with relatively preserved digit recall & WAIS scores can be used to diagnose KS.	NA	NA	NA
			Charter and Alekoumbides, 1988	KS (*n* = 15)	CG (*n* = 118; contained unclear no. with neurotic disorders or psychosis)	Memory Test Quotient (MTQ) score produced by combining the scores from Logical Memory, Visual Reproduction, & Associate Learning tests: KS < CG**^.^	MTQ misclassified 10% of participants when comparing the CG & KS group as impaired or unimpaired, respectively. The MQ misclassified 11% in the same comparison (misclassifications based on an equation proposed by Levy, 1967).	NA	Internal validity: MTQ: *r* = 0.80 MQ: *r* = 0.84.	Narrative: MTQ subtests quicker to administer than the full WMS, though could miss deficits in functions not assessed.
	WMR-R: The 2^nd^ edition of the WMS contai3ned new tests & a selection from the WMS-I, divided into 4 areas: 1 screening test (Information & orientation) which did not contribute to scoring, 3 registration tests (Mental Control, Digit Span, & Visual Memory Span), 5 encoding tests (Logical Memory 1, Figural Memory, Visual & Verbal Paired Associates 1, & Visual Reproduction 1) & 4 retention tests (Logical Memory 2, Verbal & Visual Paired Associates 2, & Visual Reproduction 2.	As For WMS-I with more visual/figural stimuli, recognition memory & delayed as well as immediate testing.	Welch et al., 1997	KS (*n* = 12) ARD (*n* = 5) Mild Alcoholic Brain Damage (MABD; *n* = 13)_r_	AL (*n* = 27) Temporal Lobe Epilepsy (TLE; *n* = 26) Parkinson's (PD; *n* = 27) Recent Toxic Exposure (RTE; *n* = 26)	Card D of Visual Reproduction (requires participants to reproduce a shape remotely resembling a sideways facing wineglass): 6 (of 30) ARCI participants reproduced a figure that more closely resembled a wineglass than the original stimulus, while no other participant did. Frequency of occurrence between combined ARCI & all other groups: χ*^2^* = 19.5^**^.	Those who produced the wineglass-like drawing had a longer history (30.5 years) & a shorter period of abstinence (*M* = 4.5 weeks) compared with the other ARCI participants (17.2 yrs drinking history; *M* abstinence = 17.8 weeks). Narrative: a query of ARCI should be raised if this type of visual confabulation is observed.	NA	NA	NA
	The 3rd edition of the WMS (-III) contained 11 subtests (6 primary, *5 optional*) which could be divided into those belonging to Auditory Memory (Logical Memory I & II, Verbal Paired Associates I & II, & *Information & Orientation, Word Lists I & II, Mental Control, & Digit Span*,) & Visual Memory (Faces I & II, Family Pictures I & II, Spatial Span, & *Visual Reproduction I & II*) scores. Combined scores termed the General Memory score. Administration time: 30-35 min for primary tests (with 15–35 min between Logical Memory I & II), plus 15–20 min for the optional subtests.	As for WMS-R with the addition of face recognition memory.	Taylor and Heaton, 2001	KS (*n* = 9)_r_	Alzheimer's Disease (*n* = 34) Huntington's (*n* = 14) Parkinson's (*n* = 9) Schizophrenia (*n* = 39) Traumatic Brain Injury (*n* = 21)	Of all the groups, KS had the lowest mean General Memory score (*M* = 58.4, *SD* = 6.4).	Sens for KS (1 *SD* from demographically-corrected mean): Auditory Memory: 100%, Visual Memory: 100%.	NA	NA	NA
			Holdnack and Delis, 2004	KS (*n* = 10)_r_	Alzheimer's Dementia (AD; *n* = 38) Huntington's Disease (*n* = 15) CG (*n* = 63)_d,i_	Face Recognition Test (FRT: Correct hits: KS < AD-HD-CG^*^ [KS vs. CG Immed/Delay: *d* = 2.07/1.76], False positives: KS-CG-AD < HD^*^ [KS vs. CG Immed/Delay: *d* = 0.03/0.81], Discriminability: KS-AD-HD < CG^*^ [KS vs. CG Immed/Delay: *d* = 1.75/1.66], Conservative bias: KS < CG-AD-HD^*^ [KS vs. CG Immed/Delay: *d* = 1.14/1.26].	Narrative: KS displayed a strong conservative response bias on the FRT (i.e., frequently saying no). In comparison, HD displayed a liberal response bias & the other groups showed no tendency in either direction. The authors stated their response bias discriminated the KS group from the other groups.	NA	NA	NA

*Tests: 3W3S, Three Words-Three Shapes; CERAD, Consortium to Establish a Registry for Alzheimer's Disease; CVLT, California Verbal-Learning Test; DPT, Doors & People Test; FCSRT, Free & Cued Selective Reminding Test; RBMT, Rivermead Behavioral Memory Test; WAIS, Wechsler Adult Intelligence Scale; WMS, Wechsler Memory Scale*.

*Samples: AL, Alcoholic group (no diagnosis of cognitive impairment); ALC, Alcoholic group with evidence of cognitive impairment not fitting KS or ARD criteria; ARD, Alcohol-Related Dementia; CG, Control Group; KS, Korsakoff's Syndrome*.

*Study quality: _r_, reference standard used to confirm diagnosis; _a_, abstinent for 6 weeks prior to testing; _d_, demographics: comparator group matched for age and gender with ARCI sample or differences accounted for in analyses; _i_, intelligence: comparator group matched for premorbid IQ and/or education or differences accounted for in analyses*.

*Outcomes: NA, not assessed*;

*, **, *** *significant at the 0.05, 0.01, and 0.001 alpha level respectively*;

ns *, not significant; d, Cohen's d effect size; η^2^, Eta squared; ROC, Receiver Operating Characteristics; AUC, Area Under Curve*.

In Duffy and O'Carroll (1994), no significant difference between KS and schizophrenic groups was observed on the Benton Visual Retention Test and Paired-Associated Learning Test, but the RBMT revealed a memory deficit “orders of magnitude” greater than the other measures, suggesting it is particularly sensitive to alcohol-related memory deficits compared with other commonly employed tests. The RBMT can also discriminate well between KS and ALs, but not between KS and ALC groups (Wester et al., 2014). However, the updated RBMT-3 appears more sensitive to less-severe memory deficits than the original and was able to significantly discriminate between an ALC and healthy control group in Wester et al. (2013b). Effect sizes indicate the most discriminating subtests appear to be those involving delayed testing and prospective memory (e.g., Messages subtest) and orientation. Compared with the original, the RBMT-3 has also been found to produce considerably less ceiling and floor effects in ARCI and control groups and classifies less healthy participants as impaired (Wester et al., 2013a). A potential shortcoming of the RBMT-3 is its failure to assess working memory and semantic memory, both of which are impaired in those with ARCI (Pitel et al., 2008; Rensen et al., 2016).

The CVLT has shown sensitivity to KS-related memory deficits across several of its variables, differentiating between those with KS, ALC, and AL groups (Brokate et al., 2003; Wester et al., 2014). Effect sizes suggest the variables most able to discriminate between these groups are the 5th learning trial of immediate testing and short and delayed free-recall. An updated version of the CVLT (CVLT-II; Delis et al., 2000) is available, though none of the studies in the review used the CVLT-II, with no rationale provided for this decision. As a result, research is needed to validate the CVLT-II in this population.

The Wechsler Memory Scale (WMS; Wechsler, 1945) in varying editions was evaluated by six studies within the review. Exploring score profiles of brain-damaged populations and controls on the WMS and its counterpart, the Wechsler Adult Intelligence Scale (WAIS), Alekoumbides et al. (1987) concluded that particularly depressed scores on the Logical Memory (LM), Visual Reproduction (VR), and Associate Learning (AL) variables of the WMS—relative to mostly preserved remaining WMS and WAIS scores—should be observed to confirm KS. The Logical Memory subtest and its variables have also shown an ability to discriminate between KS and depression, although not between KS and Alzheimer's (Kopelman, 1986). However, based on the results of multiple memory assessments, Kopelman found tests of immediate recall of small quantities of verbal information (e.g., Digit Span and immediate recall of an 8-word name and address) could effectively discriminate between the memory deficits of KS and Alzheimer's Disease, with the latter performing significantly worse.

Two response biases to WMS subtests have been identified in ARCI participants. First, after observing visual confabulations resembling a wineglass on the Visual Reproduction subtest of the WMS-R in ALs, Welch et al. (1997) searched for the same reproductions in ALs and those with ARCI. However, only six of 30 mixed ARCI participants produced the “wineglass confabulation,” suggesting its value in detecting ARCI is limited. Second, in Holdnack and Delis (2004) KS participants displayed a particularly strong negative response bias (i.e., frequently saying “no”) on the Face recognition test of the WMS-III (Wechsler, 1997), which differentiated them from those with Alzheimer's Disease and Huntington's Disease. KS performance on the test was poor overall, recognizing fewer faces than controls and the other clinical groups. The face recognition test has, however, been removed from the most recent edition of the WMS (IV), though findings are still of interest to other measures using tests of face recognition (e.g., RBMT-3).

Two tests which have demonstrated preliminary evidence of an ability to distinguish between the episodic memory deficits of those with ARCI and ALs are the Doors and People Test (DPT; Baddeley et al., 1994) and Free and Cued Selective Reminding Test (FCSRT; Buschke, 1984; Grober and Buschke, 1987). Using the cut-off point set by Maharasingam et al. (2013) of 1.5 *SD* below the norm mean, the DPT test correctly identified all KS as impaired, although also classed multiple ALs (10 of 16) as impaired. However, poor specificity at this cut-off should not be viewed as a criticism of the measure, but rather highlights its sensitivity to all alcohol-related memory deficits and suggests a further reduction in the cut-off point is required to differentiate these groups. In relation to the FCSRT, Pitel et al. (2008) have recommended the test may be useful in differentiating between KS and ALs based on finding entirely dichotomized score ranges between these groups. The FCSRT also produced a substantially large effect size between KS and AL groups (*d* = 3.54) and—by some considerable margin—the largest between KS and controls of any test reviewed (5.93).

Finally, one study evaluated the utility of the Three Words – Three Shapes (3W3S) bedside memory screening tool in those with KS. In its validation, Weintraub et al. (2000) found the 3W3S clearly differentiated the impairment of KS from controls, but not between KS and early Alzheimer's disease on any of its variables; though, a decline in memory function over repeated testing in an Alzheimer's case was reported, illustrating potential for differential diagnosis between groups. However, since its validation the 3W3S has received little further evaluation and available normative data is limited (cf. Kudiaki and Aslan, 2007).

#### Autobiographical Memory

Autobiographical memory is a form of declarative memory for personal experiences (episodic) and facts (semantic) about oneself (e.g., name, occupation). Only one study was identified which evaluated tests of autobiographical memory (Rensen et al., 2016; see Table 4). Rensen and colleagues compared the efficacy of the Autobiographical Memory Interview (AMI; Kopelman et al., 1989) and Autobiographical Interview (AI; Levine et al., 2002) in a KS and control group. The AI only asks for one memory from each recalled time-period (compared with three for the AMI), which Rensen et al. (2016) hypothesized may make it less likely to display a temporal memory gradient if there exists a particularly salient memory which has been repeatedly retrieved throughout a person's lifetime and is therefore abnormally well-preserved. However, both measures revealed a temporal gradient in memory function—a known feature of KS amnesia (Kopelman, 1989).

**Table 4:** Tests of autobiographical memory.

**Test**	**Summary**	**Aspects of memory assessed**	**Study**	**ARCI samples**	**Comparators**	**Outcomes**		
						**Between group comparisons**	**Inter-rater reliability (ICCs)**	**Practical considerations**
AI	Required to recall 1 memory from 5 life periods: childhood, adolescence, adulthood, middle age, & recent. Elements of memories are categorized as internal (episodic) or external (semantic memories & other components) Administration time: unknown.	Episodic & semantic autobiographical memory Repetitions Metacognitive statements Editorializing of memories.	Rensen et al., 2016	KS (*n* = 20; DSM-5; Kopelman et al., 2009)_r_	CG (*n* = 27)_d,i_	Internal (episodic) details: KS < CG^***^, *η_*p*_*^2^ = 0.45.Semantic details: KS < CG^*^, *η_*p*_*^2^ = 0.8.	Internal (episodic) details: *r* = 0.61. Semantic details: *r* = 0.87.	Narrative: complex scoring system.
AMI	Requires participants to recall 3 autobiographical incidents & 3 personal semantic facts from 3 life periods: childhood, young adulthood, & recent life. Administration time: 20–30 min.	Episodic & semantic autobiographical memory.				Episodic incident schedule: KS < CG^***^, *η_*p*_*^2^ = 0.60.Personal semantic schedule: KS < CG^***^, *η_*p*_*^2^ = 0.72.	Episodic incident schedule: *r* = 0.94. Personal semantic schedule: *r* = 0.99.	NA

*No normative data can be found for the AI, but normative data from a small sample using the AMI can be found in Kopelman et al. (1990)*.

*Tests: AI, Autobiographical Interview; AMI, Autobiographical Memory Interview*.

*Samples: CG, Control Group; KS, Korsakoff's Syndrome*.

*Study quality: _r_, reference standard used to confirm diagnosis; _a_, abstinent for 6 weeks prior to testing; _d_, demographics: comparator group matched for age and gender with ARCI sample or differences accounted for in analyses; _i_, intelligence: comparator group matched for premorbid IQ and/or education or differences accounted for in analyses*.

*Outcomes: NA, not assessed*;

*, *** *significant at the 0.05, 0.01, and 0.001 alpha level respectively*;

*^ns^, not significant; $\eta_{\text{p}}^{2}$, Partial Eta squared; ICCs, Intra-Class Coefficients*.

Although both assessments produced significant differences between the groups in relation to preserved episodic and semantic memory over various time-periods, a substantially larger effect size was observed for differences in semantic memory on the AMI compared with the corresponding AI variable (Rensen et al., 2016). The authors suggested this could be as the AI does not request specific semantic information like the AMI, but scores the semantic details incorporated into episodic memories. The AMI demonstrated superior inter-rater reliability, with scores in the excellent range for both the Episodic incident and Semantic personal schedules. In contrast, inter-rater reliability for the AI Semantic details score was in the good range, while the Internal (episodic) details was in the moderate range. Rensen and colleagues speculated that the complexity of the AI scoring system, even for trained raters, compared with the AMI may explain these differences.

#### Procedural Memory

Four of the included studies evaluated tools used in the assessment of procedural memory (see Table 5). Butters et al. (1985) used the Tower of Hanoi (ToH) task to assess cognitive procedural learning in KS, which they hypothesized would be preserved based on previous research showing normal performance on mirror-reading and pursuit-rotor tasks of procedural memory. Contrary to their expectations, KS participants showed significantly less learning on the ToH test over 2 days compared with controls, suggesting they were slower to acquire the necessary skills to complete the task. Butters and colleagues implicated the problem-solving element of the ToH as the cause of the KS group's poor performance—as opposed to a procedural memory deficit—and suggested the test is limited in producing an understanding of procedural learning, given that performance is also dependent on the identification, sequencing, and retention of moves. Indeed, the ToH and the modified Tower of London (ToL) and Toronto (ToT) versions are now widely employed as measures of EF, both in KS and other clinical populations.

**Table 5:** Tests of procedural memory.

**Test**	**Summary**	**Aspects of memory assessed**	**Study**	**ARCI samples**	**Comparators**	**Outcomes**			
						**Between group comparisons**	**Sensitivity & specificity**	**Validity**	**Practical considerations**
Novel Task (RBMT-3)	A newly added subtest to the RBMT series which assesses a person's ability to learn a new task by asking them to re-assemble a six-piece puzzle in the same order as the examiner, with three learning trials & a delayed testing trial. Administration time: ~5 min.	Procedural memoryWorking memoryEpisodic memoryVisuospatial processes.	(Wester et al., 2013a)	Combined KS (*n* = 15; DSM-IV-TR; Kopelman, 2002) & ALC (*n* = 10)_r,a_	CG (*n* = 25)_d,i_	Immed: ARCI < CG^***^ [*d* = 1.48], Delay: ARCI < CG^***^ [*d* = 2.82].	~50% of ARCI within the impaired range and 0% of controls.	No ceiling or floor effects on immediate trial, though half of participants (*numbers of ARCI & CG not stated*) performed at ceiling in delayed testing.	Narrative: Authors stated no other test which assess procedural learning available with normative data.
			(Wester et al., 2013b)	KS (*n* = 49; DSM-IV-TR; Kopelman, 2002)_r,a_	ALC (*n* = 49)_d,i_ CG (*n* = 53)_i_	Immed: KS < CG^***^, *d* = 1.95; KS < ALC^***^ [*d* = 0.85]; ALC < CG^*^, *d* = 0.93, Delay: KS < CG^***^, *d* = 2.67; KS < ALC^***^ [*d* = 1.33]; ALC < CG^*^, *d* = 0.81.	NA	Narrative (construct): performance may rely on spatial working memory and visuospatial episodic memory as well as procedural learning.	NA
ToH	Computerized or wooden test with 3 pegs, one with 3-5 discs ordered from largest up to smallest on a peg. Required to move the discs in a minimum no. of moves to create the same order on either of the remaining 2 pegs, only moving 1 peg at a time & without placing a large disk on top of a smaller one. Administration time: participant dependent.	Procedural learning (cognitive) Executive functions; planning, sequencing, visuospatial working memory.	Butters et al., 1985	Amnesic group (KS: *n* = 5; alternate etiology: *n* = 1)	Huntington's Disease (HD; *n* = 15) CG (*n* = 12) Post-encephalic (*n* = 1)	Improvement over time: Amnesic < CG^*^ [*d* = 1.74].	NA	Narrative (construct): 4 KS completed a mirror-reading task at normal level. Based on this & poor ToH performance, the authors concluded the ToH relies on planning & problem-solving as well as procedural learning, explaining poor KS scores.	NA
			Beaunieux et al., 1998	KS case-study (47 year old male)_r_	CG (*n* = 10)_d,i_	Time: KS-CG^ns^, No. of moves: KS-CG^ns^.	NA	Narrative (construct): ToH can better assess procedural learning if modified, though performance is still not entirely independent of working & declarative memory.	Narrative: a 4-disk version of the test results in the optimum level of difficulty for persons with brain damage.

*Tests: Novel Task (RBMT-3) = Novel Task, Rivermead Behavioral Memory Test-3; ToH = Tower of Hanoi*.

*Samples: ALC, Alcoholic group with evidence of cognitive impairment not fitting KS or ARD criteria; CG, Control Group; KS, Korsakoff's Syndrome*.

*Study quality: _r_, reference standard used to confirm diagnosis; _a_, abstinent for 6 weeks prior to testing; _d_, demographics: comparator group matched for age and gender with ARCI sample or differences accounted for in analyses; _i_, intelligence: comparator group matched for premorbid IQ and/or education or differences accounted for in analyses*.

*Outcomes: NA, not assessed*;

*, *** *significant at the 0.05, 0.01, and 0.001 alpha level respectively*;

ns *, not significant; d, Cohen's d effect size*.

It appears possible, though, that the ToH may be used to assess procedural memory by manipulating the specifics of administration. Beaunieux et al. (1998) argued that the 16 trials used in Butters et al. (1985) allowed control participants to increase their reliance on declarative memory over time, verbalizing the correct strategy for completing the task. Accordingly, they reduced the number of ToH trials per session to three, decreasing the chance of verbalization in control participants and included a pilot trial using only three (instead of four) discs, allowing participants access to the underlying problem-solving strategy required. Under these conditions, the KS participant did not differ from controls in the time-to-solve or the number of moves on the four-disk version, indicating preserved cognitive procedural learning. Still, it remains possible that this procedure was simply too easy to reveal any deficit. In those with Parkinson's Disease, impairments are not evident on easier tasks of procedural learning, but become apparent when the difficulty is increased (Haaland et al., 1997). This same hypothesis was posited by Munro et al. (2001) as an explanation for the normal performance by participants with ARD on the Pursuit Rotor Learning Task.

More recently, the RMBT-3's newly-added Novel Task subtest has been investigated as a measure of procedural learning. Wester et al. (2013a) suggested the test may rely on procedural memory for correct completion, and performance appears to be impaired in both KS and ALC groups compared to controls. Although, in a separate study, the same authors stated completion of the Novel Task may involve spatial working memory during the immediate trial and visuospatial episodic recall in delayed testing (Wester et al., 2013b), potentially explaining the poor performance on the test by those with ARCI.

#### Working Memory

Two studies evaluated tests specifically designed for working memory assessment (see Table 6). The Brown-Peterson (BP) Task (Brown, 1958; Peterson and Peterson, 1959) was employed by Leng and Parkin (1989) as a measure of working memory in those with KS, although their findings questioned the construct validity of the test. KS participants performed worse with each consecutive increase in delay length (up to 60 s). However, BP performance in the KS group was not significantly correlated with performance on any of several memory tests but was correlated with Wisconsin Card Sorting Test performance on long-delay trials. From this, Leng and Parkin suggested performance on longer BP trials may be affected not just by memory function, but also manifestations of frontal lobe damage such as perseveration—now a known feature of KS (Delis et al., 1991).

**Table 6:** Tests of working memory.

**Test**	**Summary**	**Aspects of memory assessed**	**Study**	**ARCI samples**	**Comparators**	**Outcomes**		
						**Between group comparisons**	**Sensitivity (sens) & specificity (spec)**	**Validity**
BP-Task	Participant is presented with a stimulus (typically 3 consonants or a word), after which they are asked to recall immediately or after a delay (up to 60 s), during which an interference task is undertaken (e.g., mental addition). Administration time: 10 min.	Verbal working memory Immediate & delayed recall Proactive & retroactive interference	Leng and Parkin, 1989	KS (*n* = 6)	Temporal lobe amnesia (TLA; *n* = 10) CG (*n* = 7)	All conditions: KS < TLA^***^ (*controls at ceiling – omitted from analysis*).	NA	Divergent: BP & Memory battery (comprising multiple standardized & author developed tests) correlation: *r* = −0.39^ns^.Convergent: BP & WCST: *r* = −0.60^*^.Narrative (construct): performance on long-delay trials likely recruits executive skills for correct completion.
CB-TT	Participants presented with a series of 9 blocks. The experimenter taps a series of blocks in a specific order, after which the participant is asked to tap the blocks in the same sequential order. The number of blocks in the series increases with each successful trial. Administration time: 5–10 min.	Visuospatial working memory Immediate recall	Piekema et al., 2007	KS (*n* = 15; DSM-IV; Kopelman, 2002)_r_	Temporal lobe epilepsy (TLE; *n* = 12) CG (*n* = 30)_d,i_	NA	Impaired relative to norm data (5^th^ percentile used as cut-off): 1 KS [sens = 7%].	Narrative: KS unimpaired (mostly) on CB-TT & WMS-R D-S standardized tests, but clear deficits on more difficult tasks of working memory developed by the authors. They suggested the delay period in their tests (8s) resulted in impaired KS performance.
WMS-R D-S	Participants verbally recall a series of digits in increasing numbers forwards & backwards Subtest of the WMS. Administration time: 5–10 min.	Verbal working memory Immediate recall				NA	Impaired relative to norm data (5th percentile): 2 KS [sens = 13%].	

*Tests: BP Task, Brown-Peterson Task; CB-TT, Corsi Block-Tapping Test; WMS-R D-S, Wechsler Memory Scale-Revised Digit-Span*.

*Samples: CG, Control Group; KS, Korsakoff's Syndrome*.

*Study quality: _r_, reference standard used to confirm diagnosis; _a_, abstinent for 6 weeks prior to testing; _d_, demographics: comparator group matched for age and gender with ARCI sample or differences accounted for in analyses; _i_, intelligence: comparator group matched for premorbid IQ and/or education or differences accounted for in analyses*.

*Outcomes: NA, not assessed*;

*, *** *significant at the 0.05, 0.01, and 0.001 alpha level respectively*;

ns *, not significant*.

Reviewed studies suggest the Wechsler Digit Span tests and similar assessments of immediate verbal and visual recall (e.g., Corsi-Block Tapping Test; Kessels et al., 2000) are limited in evaluating working memory of those with ARCI, whose performance is similar to controls despite clear deficits on other tasks (Harbinson, 1984; Alekoumbides et al., 1987; Leng and Parkin, 1989). To assess working memory in KS, a slight delay (~8 s) may be required to activate the maintenance component of working memory (Piekema et al., 2007).

#### Confabulation

Two studies evaluated tests of confabulation in those with ARCI (see Table 7). Rensen et al. (2015) devised the Nijmegen-Venray Confabulation List (NVCL-20) as an observational scale used to quantify confabulatory behavior and validated it in KS and ALC groups. Compared with the Dalla Barba Confabulation Battery (DBCB; Dalla Barba, 1993) and the Provoked Confabulation Test (PCT; Cooper et al., 2006), the scale demonstrated superior discriminatory ability between the two samples and good to excellent internal reliability. Good to excellent inter-rater reliability was also found between primary and secondary caregivers (excluding the Provoked Confabulation category scores for the KS group, which scored moderately), though the participants were divided according to their diagnoses as presumed confabulators (KS) and non-confabulators (ALC) and raters were not blind to diagnosis, potentially confounding between-rater results.

**Table 7:** Tests of confabulation.

**Test**	**Summary**	**Study**	**ARCI samples**	**Comparators**	**Outcomes**					
					**Between group comparisons**	**Convergent & divergent validity**	**Concurrent validity**	**Inter-rater reliability (ICCs)**	**Internal consistency (Lambda 2)**	**Practical considerations**
CVLT-I	An index of word intrusions divided into semantically related (i.e., to the information learned) & unrelated intrusions–used as an index of confabulation. CVLT administration time: 20–50 min.	Rensen et al., 2017	KS (*n* = 51; DSM-5; Kopelman et al., 2009)_r_	–	–	No sig. correlations between spontaneous or provoked confabulation & related intrusions or unrelated intrusions.	NA	NA	NA	NA
DBCB	Semi-structured interview containing 64 questions divided into 7 categories. Administration time: ~30 min.	Rensen et al., 2015. Varying sample sizes used for analyses	KS (*n* = 28; DSM-5; Kopelman, 2002)_r,a_	ALC (*n* = 24; DSM-5 mild neurocognitive disorder due to alcohol)_d,i_	“*I don't know*” episodic: KS < ALC^*^ [*d* = 0.66]. All 8 other variables: KS-ALC^ns^.	Confabulation score: No sig. correlations with any measures of memory or EF.	Correlations with DBCB Confabulation/ Total correct: PCT confabulation: *r* = 0.114^ns^/ −0.096^ns^ PCT Total Score: *r* = −0.241/ 0.466^*^.	NA	NA	NA
NVCL-20	A 20-question observational scale completed by a caregiver. Produces 3 category scores – Spontaneous confabulation, Provoked confabulation, & Memory & Orientation (M & O) – & a Total Confabulation Score. Questions 8, 9, & 11 contribute to the total score, but none of the category scores. Completion & scoring takes ~10–20 min.				All four variables: KS < ALC^***^ [Total score: *d* = 1.06, Spontaneous confabulation: *d* = 0.75, Provoked confabulation: *d* = 1.19, & Memory & Orientation: *d* = 1.05].	Total score: Significantly correlated with scores on the RBMT-3^*^ & CVLT total score^*^ (but not CVLT Intrusions). No sig. correlations with any measures of EF.	Correlations with NVCL-20 Spontaneous/ Provoked/ Memory & Orientation/ Total Score: PCT Confabulation: *r* = 0.478^***^/ 0.394^*^/ 0.400^*^/ 0.506^**^; PCT Total: *r* = −0.519^**^/ −0.528^**^/ −0.531^**^/ −0.569^**^; DBCB Confabulation: *r* = 0.008^ns^/ 0.151^ns^/ / 0.260^ns^/ 0.173^ns^; DBCB Total: *r* = −0.247^ns^/ −0.393^*^/ −0.403^*^/ −0.441^*^.	KS/ ALC/ Combined: Spontaneous = 0.79/ 0.64/ 0.78 Provoked = 0.50/ 0.62/0.62 Memory & Orientation = 0.77/ 0.73/ 0.80 Total score = 0.76/ 0.68/ 0.79.	KS/ALC/ Combined: Spontaneous = 0.91/ -^#^/0.91 Provoked = 0.75/ 0.83/ 0.91 Memory & Orientation = 0.91/ 0.90/ 0.81 Total score = 0.94/ 0.95/ 0.96.	Narrative: can be completed by professional caregivers or relatives. Available for free from the corresponding author.
PCT	Participants are asked to name 5 picture cards (e.g., a football player) then construct a short story based on the images. After a short delay, they are then asked to freely recall the story, followed by a recognition task related to the images. Administration time: unknown.				Both variables: KS-ALC^ns^.	Confabulation score: No sig. correlations with any measures of memory or EF.	–	NA	NA	NA

*Tests: CVLT-I, California Verbal Learning Test – Intrusions variable; Dalla Barba Confabulation Battery; NVCL-20, Nijmegen-Venray Confabulation List; PCT, Provoked Confabulation Test*.

*Samples: ALC, Alcoholic group with evidence of cognitive impairment not fitting KS or ARD criteria; KS, Korsakoff's Syndrome*.

*Study quality: _r_, reference standard used to confirm diagnosis; _a_, abstinent for 6 weeks prior to testing; _d_, demographics: comparator group matched for age and gender with ARCI sample or differences accounted for in analyses; _i_, intelligence: comparator group matched for premorbid IQ and/or education or differences accounted for in analyses*.

*Outcomes: NA, not assessed*;

*, **, *** *significant at the 0.05, 0.01, and 0.001 alpha level respectively*;

ns *, not significant; d, Cohen's d effect size; ICCs, Intra-Class Coefficients*.

# *Internal consistency could not be calculated for the ALC group on the Spontaneous Confabulations category due to floor-effects*.

The NVCL-20 total score, unlike the DBCB and PCT, correlated significantly with RMBT-3 and CVLT scores in KS (Rensen et al., 2015). Thus, those who performed poorly on memory tests tended to score higher on the NVCL-20 (indicating greater confabulation). Conversely, no significant correlation was found between the NVCL-20 and the CVLT intrusions variable, questioning the previous use of this as an index of confabulation (e.g., Schnider et al., 1996). Rensen et al.'s (2017) findings corroborate this concern, finding no correlation between spontaneous and provoked confabulation as measured by the NVCL-20 and the total number of intrusions on the CLVT. However, small but non-significant correlations were found between unrelated intrusions and both forms of confabulation (*r* = 0.20 for both analyses), suggesting intrusions unrelated to the information learnt may be more closely associated with confabulations than those related.

### Executive Function

Six studies evaluated tests of EF, all in KS participants (see Table 8).

**Table 8:** Tests of executive function.

**Test**	**Summary**	**Functions assessed**	**Study**	**ARCI samples**	**Comparators**	**Outcomes**				
						**Between group comparisons**	**Sensitivity (sens) & specificity (spec)**	**Validity**	**Reliability**	**Practical considerations**
BADS	Test battery designed to measure various executive functions, divided into 6 subtests scored on a range of 0-4, with a total maximum profile score of 24. Profile scores can be converted to standardized scores with a mean of 100 & SD of 15. Also includes a dysexecutive questionnaire which does not contribute to the profile score but provides supplemental qualitative information about a person's executive deficits. Administration time: 30 min.	Concept shifting Response inhibition Planning Problem solving Self-monitoring Reasoned estimation Strategy use Unstructured planning Organization Set shifting.	van Oort and Kessels, 2009	KS (*n* = 20; DSM-IV-TR)_r,a_	–	–	All cut-offs 1.5 *SD* below the norm: 8 KS impaired on Total Score. Most impaired on Six Elements (*n* = 11) & least on Temporal Judgment (*n* = 1).	Narrative (content): the tool assesses multiple executive abilities.	NA	NA
			Maharasingam et al., 2013	KS (*n* = 15; Caine et al., 1997)_r_	AL (*n* = 16)_d,i_	Rule Shift: KS < AL^***^, η^2^ = 0.41 [*d* = 1.69]; Temporal judgment: KS-AL^ns^; Action Program: KS < AL^*^, η^2^ = 0.20 [*d* = 3.40]; Key Search: KS-AL^ns^; Zoo Map: KS < AL^*^, η^2^ = 0.18 [*d* = 0.94]; Modified Six Elements: KS < AL^***^, η^2^ = 0.40 [*d* = 6.0].	Total age-corrected Score (cut-off: 1.5 *SD* below mean): 13 KS & 2 AL classed as impaired [sens = 87.5%, spec = 86.6%].	NA	NA	NA
BSPAT	Participants shown 56 pages, each with 10 circles on, 1 of which is colored blue. The position of the blue dot changes on each page following a simple rule. Participants are asked to suggest where the dot will be on each following page. Every error is scored, with a maximum of 55 points. Administration time: 10 min.	Rule detection & following Ability to change strategy in the face of new information.	Van Den Berg et al., 2009	KS (*n* = 41; Kopelman, 2002)_r_	Psychiatric disorders (*n* = 63) Stroke (*n* = 106) Diabetes Mellitus (*n* = 376) MCI/early dementia (*n* = 70) CG (*n* = 282)_d,i_	Total errors: KS> CG^***^ [*d* = 0.083]. Higher percentage of KS in impaired range (*n* = 12; 29%) & below average range (*n* = 28; 16%) than all other groups.	KS vs. CG: ROC AUC = 0.74^***^ (95% CI = 0.66–0.82). Optimal cut-off >18/19 errors: sens = 81%, spec = 62%.	NA	CG (*n* = 83): test-re-test: *r* = 0.61^***^.	Narrative: does not require verbal or complex motor response. Relatively short administration time makes it a useful addition to testing.
CET	Participants asked to produce estimations to 15 questions where the actual answer is unlikely to be known, but a reasonable estimate can be made based on general knowledge (e.g., What is the length of the average man's spine?). Responses scored on a 4-point scale, with greater scores indicating poorer performance. Shoqeirat et al. (1990) have produced a shortened version using 9 questions from the CET & the WAIS population estimation question. Administration time: 10-20 min.	Estimations of quantity, time, distance & weight Problem solving Semantic memory.	Kopelman, 1991	KS (*n* = 16)_r,a_	Alzheimer's Dementia (AD; *n* = 16)_d,i_ CG (*n* = 16)_d,i_	NA	NA	Convergent (KS): FAS: *r =* −0.20^ns^. MCST categories achieved: *r* = −0.27^ns^, MCST % perseverative errors: *r* = 0.10^ns^. KS & AD: NART: *r* = 0.40^*^, WAIS FSIQ: *r* = 0.53*^**^.	NA	NA
			Taylor and O'Carroll, 1995	KS (*n* = 18; DSM-III-R)_r_	Nine mixed neuro-psychiatric groups including discrete frontal lesions (all groups: MNP; *n* = 352) CG (*n* = 150)_i_	Shortened CET: KS>CG^*^ [*d* = 1.22], CG-frontal lesion group^ns^, CG-all other MN groups^ns^.	NA	Narrative: estimations rely on unique skills impaired in KS, such as retrieval from semantic memory & error checking of this.	NA	NA
FAS	Test of verbal fluency requiring participants to state as many words as they can which begin with F, A, & S, each within a time limit of 1 min. Administration time: 5 min.	Phonemic verbal fluency Semantic & working memory.	Shoqeirat et al., 1990	KS (*n* = 16)	Herpes simplex encephalitis amnesiacs (HSE; *n* = 10) Anterior communicating artery aneurysm amnesiacs (ACAA; *n* = 5) CG (*n* = 31)_i_	KS-HSE^ns^, KS-ACCA^ns^, KS < CG^*^ [*d* = 0.77].	NA	Convergent: CET (shortened): *r* = −0.34^ns^.	NA	NA
			Kopelman, 1991	KS (*n* = 16)_r,a_	Alzheimer's Dementia (AD; *n* = 16)_d,i_ CG (*n* = 16)_d,i_	NA	NA	Convergent (KS): MCST categories achieved: *r* = 0.63^**^, MCST % perseverative errors:	NA	
								*r* = −0.46^*^. KS & AD: NART: *r* = 0.44*, WAIS FSIQ: *r* = 0.45^**^.		
WCST	Participants presented with 4 stimulus cards which display different geometric shapes in varying numbers & colors. Next, 2 packs of 64 response cards are given & the person is asked to match these to 1 of the 4 stimulus cards, after which they are told whether they were correct. The rule that determines how to correctly match cards changes without warning. The test has been adapted by Nelson (1976) to create the Modified Card Sorting Test (MCST), made easier than the WCST by the shortening of the test (2 packs of 24 card), the removal of ambiguous stimuli, requiring fewer consecutive sorts to achieve a category, & informing participants upon changes in category. Administration: 15–30 min.	Rule detection & following Ability to change strategy in the face of new information Perseveration.	Shoqeirat et al., 1990	KS (*n* = 16)	Herpes simplex encephalitis amnesiacs (HSE; *n* = 10) Anterior communicating artery aneurysm amnesiacs (ACAA; *n* = 5) CG (*n* = 31)_i_	Full WCST: Categories achieved: KS < HSE^**^ [*d* = 1.35.], KS-ACCA^ns^, KS < norm data**. Perseveration: KS> HSE^**^ [*d* = 1.60], KS-ACCA^ns^, KS> norm data**. MCST: Categories achieved: KS < HSE^*^ [*d* = 1.30], KS-ACCA^ns^ [*d* = 0.65], KS < CG^**^ [*d* = 1.39]. Perseveration & total errors: KS-HSE^ns^, KS-ACCA^ns^, KS> CG^*^ [*d* = 0.94/1.11].	Narration: authors concluded the MCST is less sensitive than the WCST to executive deficits & therefore less able to discriminate between amnesiac groups.	Convergent: correlation between all W/MCST variables & CET (shortened) scores^ns^. Correlation between all WCST & MCST variables & FAS scores^ns^.	NA	Narrative: Nelson's MCST quicker than the WCST to administer, though less sensitive.
			Kopelman, 1991	KS (*n* = 16)_r,a_	Alzheimer's Dementia (AD; *n* = 16)_d,i_ CG (*n* = 16)_d,i_	NA	NA	Convergent (KS & AD; MCST): Categories & WAIS FSIQ: *r* = 0.53***; % perseverations & WAIS FSIQ = −0.35*; % perseverations & NART = −0.44^**^.	NA	NA

*Tests: BADS, Behavioral Assessment of the Dysexecutive Syndrome; BSPAT, Brixton Spatial Anticipation Test; Cognitive Estimation Test; FAS, FAS Verbal Fluency Test; MCST, Modified Card Sorting Test; WAIS, Wechsler Adult Intelligence Scale; WCST, Wisconsin Card Sorting Test*.

*Samples: ARBD, Alcohol-Related Brain Damage; AL, Alcoholic group (no diagnosis of cognitive impairment); ARD, Alcohol-Related Dementia; CG, Control Group; MCI, Mild Cognitive Impairment; KS, Korsakoff's Syndrome*.

*Study quality: _r_, reference standard used to confirm diagnosis; _a_, abstinent for 6 weeks prior to testing; _d_, demographics: comparator group matched for age and gender with ARCI sample or differences accounted for in analyses; _i_, intelligence: comparator group matched for premorbid IQ and/or education or differences accounted for in analyses*.

*Outcomes: NA, not assessed*;

*, **, *** *significant at the 0.05, 0.01 and 0.001 alpha level respectively*;

ns *, not significant; d, Cohen's d effect size; η^2^, Eta squared; ROC, Receiver Operating Characteristics; AUC, Area Under Curve*.

Two studies found evidence for the sensitivity of the ecologically-based Behavioral Assessment of the Dysexecutive Syndrome (BADS; Wilson et al., 1996) to KS-related impairments. When comparing KS and ALs, the Six Elements and Rule Shift sub-tests have produced particularly large effect sizes (η^2^), approaching those observed on memory testing (Doors & People; Maharasingam et al., 2013). Indeed, when converted to Cohen's *d* for comparison with the other effect sizes calculated here, the Six Elements effect size (KS vs. AL) was the largest of any observed in the review (*d* = 6.0; Maharasingam et al., 2013). When employing the BADS it is important to observe individual sub-test scores as only 40% of KS were classed as impaired on the overall age-corrected score in van Oort and Kessels (2009), although this figure rose to 87% in Maharasingam et al. (2013).

The Wisconsin Card Sorting Test (WCST; Heaton et al., 1993) appears highly sensitive to KS-related EF deficits (Shoqeirat et al., 1990). A shortened and simplified version of the WCST, the Modified Card Sorting Test (MCST; Nelson, 1976), also appears sensitive, although to a lesser extent. Comparing KS performance on both the full WCST and Nelson's version, Shoqeirat et al. (1990) found the WCST to be more sensitive to severe executive dysfunction and therefore better able to discriminate between KS and other amnesic groups. The authors stated that this cannot simply because the full WCST is harder (suggesting differences in test procedures are responsible) and advocate investing the additional time required to employ the full version over the MCST. A third rule derivation test which has been used to assess ARCI is the Brixton Spatial Anticipation Test (BSPAT). In a relatively large sample of KS (*n* = 41) participants, Van Den Berg et al. (2009) found the BSPAT demonstrated good sensitivity and specificity when discriminating between KS and controls. The measure may be particularly sensitive to the executive deficits associated with ARCI as the KS group performed significantly worse than multiple other cognitively impaired groups.

Tests of cognitive estimations are also impaired in those with KS (Shoqeirat et al., 1990; Kopelman, 1991), although whether this reflects executive dysfunction remains uncertain. Impaired Cognitive Estimation Test (CET) performance in those with KS may also result from semantic memory impairments and a failure to “error check” the estimation (Taylor and O'Carroll, 1995). Moreover, despite impairments on the CET, the cognitive estimation component of the BADS (i.e., Temporal Judgement) appears the least sensitive subtest to KS (Van Den Berg et al., 2009; Maharasingam et al., 2013); though, this may be due to the use of only four questions, compared with 10-15 in the CET.

Two reviewed studies examined the inter-correlations of tests of single EFs in those with ARCI in attempts to see where scores converged or diverged. The CET does not appear to correlate with the WCST, MCST, or FAS verbal fluency test (Shoqeirat et al., 1990; Kopelman, 1991), and moderate correlations have been observed between the MCST and FAS (Kopelman, 1991), but not consistently and not between the WCST and FAS (Shoqeirat et al., 1990). The absence of correlations likely reflects the distinct executive skills used to complete these diverse tasks.

### Intelligence and Test Batteries

In total, ten of the studies investigated intelligence or battery tests (see Table 9). The Wechsler Adult Intelligence Scale (WAIS; Wechsler, 1958) was investigated in its varying editions by nine studies, often in conjunction with its counterpart, the WMS. The WAIS-WMS combination brings multiple benefits in terms of its extensive assessment of cognition, shared norms, and comparisons both within and between the tests. Though, Taylor and Heaton (2001) found 47% of the normative sample scored within the impaired range on at least one factor score (e.g., Working Memory, Verbal Comprehension etc.) from the combined Wechsler battery and 14% were impaired on at least two, indicating poor specificity. This highlights the need to identify a disease-specific profile for ARCI using this test combination. Historically, this has come in the form of a substantially impaired WMS score compared with WAIS Full-Scale IQ (FSIQ) for KS. The discrepancy between these scores has been found to be greater for KS than ALs, those with various neuropsychiatric disorders, and controls (Oscar-Berman et al., 1993; Taylor and Heaton, 2001). However, impaired FSIQ in those with ARCI has also been found, primarily due to poor performance on tests within the Performance IQ (PIQ) component (Taylor and Heaton, 2001). As a result, the discrepancy between the relatively preserved Verbal IQ (VIQ) component of the WAIS and the MQ may be a more effective indicator of KS (Alekoumbides et al., 1987; Charter and Alekoumbides, 1988). However, caution should be exercised in using these discrepancies for the diagnosis of ARCI for reasons outlined in the discussion.

**Table 9:** Tests of intelligence and test batteries.

**Test**	**Summary**	**Functions assessed**	**Study**	**ARCI samples**	**Comparators**	**Outcomes**			
						**Between group comparisons**	**Sensitivity (sens) & specificity (spec)**	**Validity**	**Practical considerations**
CANTAB	Computerized assessment comprising 2 screening tests designed to detect visual, motor & comprehension problems that may confound performance & 22 primary tests of various cognitive abilities. Administration time: 3 min for screening, 3–10 min for primary tests (mean = ~7 min).	Reaction time Visual episodic & working memory Verbal/semantic memory Sustaining & shifting attention Impulse control Risk taking Spatial planning Emotion recognition Rule acquisition.	Horton et al., 2015b	ARBD (*n = 19–16 due to attrition)_*a*_*	–	–	*Z* scores indicate varying numbers of KS impaired relative to norm data across multiple 5 subtests. Most impaired on Paired Associated Learning (50–65%) & least on Spatial Working Memory (5%). Narrative: floor effects observed on the Rapid Visual Information Processing test.	NA	Narrative: requires little training to administer. Use of parallel versions allows repeated testing without learning effects. Absence of normative data for some tests is limiting. [3 KS withdraw from participation−1 each following 2, 3, & 4 tests].
WAIS	WAIS-I: the original Wechsler intelligence scale comprised 9 subtests which contributed to either a Performance IQ (PIQ) or Verbal IQ (VIQ) score, which when combined produced a Full-Scale IQ (FSIQ) score. The FSIQ has a standardized mean of 100 & *SD* of 15, allowing direct comparison with the co-normed Wechsler Memory Scale scores. The tests belonging to the PIQ included Picture Completion (PC), Block Design (BD), Object Assembly (OA) & the Digit Symbol Substitution Test (DSST). Tests within the VIQ were Comprehension (C), Arithmetic (A), Similarities (S), Digit-Span (D-S) & Vocabulary (V).	Verbal knowledge & reasoning. Non-verbal reasoning Processing speed Working memory.	Glosser et al., 1977	KS (*n* = 10)	AL (*n* = 10) Right hemisphere pathology (*n* = 10) CG (*n* = 10)	DSST: Items completed: KS < AL-CG^*^, Processing speed: KS > AL, CG^*^, Errors: KS, AL, CG^ns^.	NA	Narrative (construct): DSST performance dependent on visuospatial processing & psychomotor speed in AL & KS, but mostly only the latter in controls.	NA
			Kapur and Butters, 1977	KS (*n* = 10)	AL (*n* = 12)_i_ CG (*n* = 10)_i_	DSST (Items completed): KS < CG^***^ [*d* = 1.99], AL < CG^*^ [*d* = 0.92], [KS vs. AL: *d* = 0.46, no significance testing done].	NA	Convergent: KS DSST performance correlated with an embedded figures test (*r* = 0.68^*^), but not paired associated learning (*r* = −0.04). Narrative (construct): poor DSST performance in KS due to an inability to learn new associations & impaired visuospatial processing, but in CG dependent on the speed of learning & subsequent psychomotor speed.	NA
			Harbinson, 1984	KS (*n* = 10; ICD)_r_	CG (*n* = 9)_d,i_	PIQ, FSIQ, DSST, Block Design, & Object Assembly: KS < CG^***^ VIQ, Arithmetic & Picture Completion: KS < CG^**^ Comprehension, Digit-Span, & Vocabulary: KS-CG^ns^.	Narrative: authors stated the DSST test was a particularly useful & sensitive indicator of KS which significantly correlated with CT scan indices of temporal lobe atrophy & electroencephalogram P3 latency.	NA	NA
			Alekoumbides et al., 1987	KS (*n* = 15)	CG (*n* = 118; contained unclear no. with neurotic disorders or psychosis)	Information: KS < CG** Comprehension, VIQ, & FSIQ: KS < CG* All other subtests: KS-CG^ns^.	Narrative: a lowered Memory Quotient (MQ) from the WMS relative to VIQ, PIQ or FSIQ is indicative of KS. The MQ-PIQ discrepancy was more sensitive to KS than PIQ-VIQ.	NA	NA
			Mazzucchi et al., 1987	ARD (*n* = 27)	Depression (*n* = 17)_i_ Alzheimer's Dementia (AD; *n* = 72)_i_ Multi-Infarct Dementia (MID; *n* = 18)_i_	Block Design: ARD < Depression^*^ (theta value >0.138) Remaining subtests: ARD < Depression^ns^ All subtests: ARD-AD-MID^ns^.	Discriminant analysis found the WAIS was not useful in differentiating between dementia types, but was useful in differentiating dementias from depression, though only the PIQ tests (particularly Block Design for ARD).	NA	NA
	WAIS-R: revised version with 11 subtests, including 9 from the WAIS-I & 2 new tests: Information (I) & Picture Arrangement (PA). Tests again belonged to either the PIQ (PC, BD, Digit Symbol, PA, OA) or VIQ (C, A, S, D-S, V, I).	As for WAIS-I with a test of general knowledge (Information) & an additional test of visual processing speed (Picture Completion)	Deary et al., 1991	KS (*n* = 11)_r,a_	Alzheimer's Dementia (AD; *n* = 10)_i_ CG (*n* = 11)_i_	Digit Symbol (DS): AD < KS^**^ [*d* = −1.29], KS < CG^***^ [*d* = 2.01].	NA	Convergent: KS: DS & MMSE: *r* = 0.69; KS: DS & CAMCOG: *r* = 0.83; KS & AD: DS & MMSE: *r* = 0.80; KS & AD: DS & CAMCOG: *r* = 0.86.	NA
			Oscar-Berman et al., 1993	KS (55–71 years; *n* = 27)_r_	Young AL (YAL; 25–48 years; *n* = 8) Older AL (OAL; 51–72 years; *n* = 15)_d_ Young CG (YCG; 22–49 years; *n* = 14) Older CG (OCG; 50–74 years; *n* = 8)_d_	WAIS VIQ: KS < OCG**, KS < OAL* WAIS-R VIQ: KS < OCG**, KS < OAL** WAIS-R PIQ: KS < OCG** WAIS FSIQ: KS < OCG* WAIS-R FSIQ: KS < OCG**, KS < OAL* All other comparisons of the same variables: KS, OLA, OCG^ns^.	WAIS-I VIQ – WMS-I MQ discrepancy: KS>OCG-OAL* WAIS-R VIQ – WMS-R GM discrepancy: KS> OCG-OAL* WAIS-I FSIQ – WMS-I MQ discrepancy: KS> OCG-OAL* WAIS-R FSIQ – WMS-R GM discrepancy: KS-OCG-OAL^ns^.	NA	NA
			Oscar-Berman et al., 2004	KS (*n* = 6)_r_	AL (*n* = 50)_i_ Right Hemisphere Lesions (RH; *n* = 6) CG (*n* = 82)_i_	Digit Symbol (DS): KS < AL^**^ [*d* = 1.77], KS < CG^**^ [*d* = 2.19], AL < CG^*^ [*d* = 0.44], KS-RH^ns^.	Authors employed various tests of memory (e.g., WMS), executive function (e.g., WCST) & visuospatial function (e.g., DS). In ALs, DS scores were the only to significantly differ from controls with up to 5-years abstinence. DS scores also significantly poorer in ALs with greater consumption & shorter abstinence.	NA	NA
	WAIS-III: contained 14 tests (2 optional), 11 from the WAIR-R & 3 new (Matrix Reasoning [MR], Symbol Search [SS], Letter-Number Sequencing [L-NS]). PIQ further divided into two factors: Perceptual Organization (PC, BD, MR) & Processing Speed (Digit-Symbol Coding, PA, OA, SS). VIQ also divided into two factors: Verbal Comprehension (V, S, I, C) & Working Memory (A, D-S, L-NS). 50–60% of WAIS-R items retained in original or marginally modified form. Administration time: 60–90 min.	As for the WAIS-R with the additional focus on working memory & processing speed (Letter-Number Sequencing, Symbol Search), & fluid non-verbal reasoning (Matrix Reasoning).	Taylor and Heaton, 2001	KS (*n* = 9)_r_	Alzheimer's Disease (AD; *n* = 34) Huntington's (HD; *n* = 14) Parkinson's (PD; *n* = 9) Schizophrenia (SC; *n* = 39) Traumatic Brain Injury (TBI; *n* = 21)	Of all the groups, the KS had the highest age-corrected FSIQ (*M* = 93.7, *SD* = 14.1) but the lowest WMS General Memory (GM) score (*M* = 58.4, *SD* = 6.4) [IQ-GM discrepancy: KS = −35.4, AD = −25.5, HD = −6.5, PD = −6.6, SC = −6.3, TBI = −4.8].	All clinical groups: 1 *SD* from demographically corrected mean produced the best sens-spec balance. Sens for KS sample (1 *SD* from corrected mean): Perceptual organization = 22.2%; Processing Speed = 55.6%; Verbal Comprehension = 22.2%; Working Memory = 11.1%. Percentage of normative sample classified as impaired (1 *SD* from corrected mean) on WAIS & WMS-III factor scores (e.g., Perceptual Organization, Working Memory): 21% impaired on 1 factor, 11% on 2, 7% on 3, 4% on 4, 2% on 5, 1% on 6 (47% impaired on at least 1).	NA	NA

*Tests: CANTAB, Cambridge Automated Neuropsychological Test Battery; WAIS, Wechsler Adult Intelligence Scale*.

*Samples: ARBD, Alcohol-Related Brain Damage; AL, Alcoholic group (no diagnosis of cognitive impairment); ARD, Alcohol-Related Dementia; CG, Control Group; KS, Korsakoff's Syndrome*.

*Study quality: _r_, reference standard used to confirm diagnosis; _a_, abstinent for 6 weeks prior to testing; _d_, demographics: comparator group matched for age and gender with ARCI sample or differences accounted for in analyses; _i_, intelligence: comparator group matched for premorbid IQ and/or education or differences accounted for in analyses*.

*Outcomes: NA, not assessed*;

*, **, *** *significant at the 0.05, 0.01, and 0.001 alpha level respectively*;

ns *, not significant; d, Cohen's d effect size (minus values indicate the ARCI group performed better than comparator)*.

The specific WAIS tests on which KS have demonstrated impaired performance include the Digit Symbol Substitution Test (DSST), Block Design, Picture Completion and Object Assembly (Malerstein and Belden, 1968; Glosser et al., 1977; Harbinson, 1984; Oscar-Berman et al., 2004). Of these, the DSST and its replacements have shown the greatest sensitivity to ARCI (Glosser et al., 1977; Harbinson, 1984) and have consistently differentiated KS from ALs, who are (to a lesser extent) also impaired on the test (Oscar-Berman et al., 2004). DSST performance appears to depend on a combination of new learning (Kapur and Butters, 1977), psychomotor speed, and visuospatial processing (Glosser et al., 1977), explaining its sensitivity to ARCI.

The CANTAB computerized battery assessment was investigated by one of the included studies. Horton et al. (2015b) found those with ARBD were impaired relative to norm scores on various CANTAB tests, particularly the Rapid Visual Processing (RVP) test of sustained visual attention; though the authors suggest this may reflect a processing speed deficit rather than one of attention. Floor effects were also observed on the RVP, suggesting this test may be limited in assessing the spectrum of ability in those with ARCI. However, the application of Horton and colleagues' findings is restricted as they failed to clearly outline the definition of ARBD they adopted and the criteria by which this diagnosis was made. They also omitted whether their sample comprised individuals with differing diagnostic labels (e.g., WE, KS, ARD, and/or ALC), as has been reported by other studies of participants with “ARBD” (e.g., Wilson et al., 2012).

### Premorbid Ability

Three studies investigated methods of estimating premorbid ability (all attempted to predict IQ) in those with ARCI (see Table 10), providing some insight into the value of the three approaches typically used: [1] predictions based on demographics (e.g., education, gender, ethnicity), [2] current performance on tasks believed to be relatively impervious to neurological damage, and [3] a combination of these factors. Demographically based predictions may be useful when assessing individuals with ARCI as no testing is required and thus estimates are not influenced by participant effort or the effects of alcoholism and associated factors (e.g., head injuries). O'Carroll et al. (1992) found demographically based estimates strongly correlated with those derived by the National Adult Reading Test (NART) of premorbid IQ. However, Bright et al. (2002) found NART and NART-R based estimates accounted for substantially more of the variance in current WAIS FSIQ than demographic variables, suggesting the NART may be the more accurate predictor.

**Table 10:** Tests of premorbid function.

**Test**	**Summary**	**Study**	**ARCI samples**	**Comparators**	**Outcomes**	
					**Between group comparisons**	**Validity**
NART	Requires participants to read & orally pronounce 50 phonetically irregular words. Performance is expressed as the number of words incorrectly pronounced & scores can be transformed into WAIS-FSIQ estimates. Performance is assumed to be relatively unaffected by varying types & severities of neurological damage. Administration time: 10–15 min.	Crawford et al., 1988	KS (*n* = 12; DSM-III)_r_ ARD (*n* = 12, DSM-III)_r_	Each ARCI participant demographically matched to a control (*n* = 70 in total)_d,i_	Estimated IQ: KS < CG^*^ ARD-CG^ns^.	Predictive: NART predicted a significantly higher estimation of IQ than the WAIS-VO in both KS^*^ & ARD^*^.
		O'Carroll et al., 1992	KS (*n* = 20)_r,a_	CG (*n* = 40)_d,i_	Errors: KS>CG^***^ [*d* = 2.03].	Convergent: NART & demographically predicated premorbid IQ: *r* = 0.74^***^. Narrative (construct): depressed KS score relative to demographically predicated IQ suggested their NART performance was dependent on factors other than vocabulary.
	NART-R: Revised version re-standardized for the WAIS-R. Administration time: 10–15 min.	Bright et al., 2002	KS (*n* = 35; Cutting, 1978 criteria)_r_	CG (*n* = 51)_d,i_	Estimated IQ: NART: KS-CG^ns^ NART-R: KS-CG^ns^.	Convergent (KS): NART & NART-R: *r* = 1.0^***^; NART & WAIS: *r* = 0.77^***^; NART & WAIS-R: *r* = 0.72^***^.CG: NART & NART-R better predictors of actual WAIS-III FSIQ (50% of variance) than estimates based on demographic variables (25%).
WAIS-VO	Participants asked to provide oral definitions for multiple words. Administration time: 10 min.	Crawford et al., 1988	KS (*n* = 12; DSM-III)_r_ ARD (*n* = 12, DSM-III)_r_	Each ARCI participant demographically matched to a control (*n* = 70 in total)_d,i_	Estimated IQ: KS < CG^**^ ARD < CG^*^.	Predictive: WAIS-VO predicted a significantly lower estimation of IQ than the NART in both KS & ARD^*^.

*Tests: NART, National Adult Reading Test; WAIS VO, Wechsler Adult Intelligence Scale, Vocabulary Subtest*.

*Samples: ARD, Alcohol-Related Dementia; CG, Control Group; KS, Korsakoff's Syndrome*.

*Study quality: _r_, reference standard used to confirm diagnosis; _a_, abstinent for 6 weeks prior to testing; _d_, demographics: comparator group matched for age and gender with ARCI sample or differences accounted for in analyses; _i_, intelligence: comparator group matched for premorbid IQ and/or education or differences accounted for in analyses*.

*Statistical: NA, not assessed*;

*, **, *** *significant at the 0.05, 0.01, and 0.001 alpha level respectively*;

ns *, not significant; d, Cohen's d effect size*.

Crawford et al. (1988) have recommended the use of the NART in those with ARD after finding little difference between ARD NART scores and those of demographically matched (age, gender, education) controls. The authors also, however, suggested the NART may be unsuitable for use with KS as it produced a significantly lower IQ than was estimated for a matched control group. Nonetheless, Crawford et al. (1988) advocated the NART for use with KS over the WAIS Vocabulary test, which produced lower estimates again. O'Carroll et al. (1992) have also questioned the validity of using the NART with KS based on finding its estimates were significantly below those of demographically-derived predictions. Drawing on their findings and those of Crawford and colleagues, O'Carroll et al. (1992) speculated the impaired NART performance in KS may result from executive dysfunction, suggesting that individuals with KS will quickly read aloud the words according to standard phonetical rules of pronunciation, without the necessary error checking process required for irregular word pronunciation. However, the processes underlying impaired NART performance in KS are yet to be fully elucidated, and those with frontal lesions appear to perform comparably with matched controls (Bright et al., 2002; MacPherson et al., 2017), questioning the role of executive dysfunction.

In contrast to both Crawford et al. (1988) and O'Carroll et al. (1992), Bright et al. (2002) found no significant difference between a larger sample of KS participants and matched controls on both the NART and NART-R. Moreover, the NART-R produced a smaller discrepancy between its scores and those derived from demographic variables than the original NART, indicating an improvement in predictive accuracy. Finally, Bright et al. found no additional benefit of combining NART scores with demographic variables to better estimate premorbid function in KS, other various neuropsychiatric disorders or controls. However, some value in combining these approaches has been documented in the wider literature (e.g., Vanderploeg and Schinka, 1995).

### Assessment of Study Quality

The summarized results of the study quality appraisal are presented in Table 11 (see Supplemental Document 1 for a tabulated overview of the entire quality assessment outcome). The quality assessment of studies revealed that the description of ARCI samples was frequently poor, with many authors failing to state whether they were abstinent, if they were diagnosed according to nosological criteria (e.g., DSM or ICD), how they were diagnosed, and whether those with complicating comorbid disorders (e.g., dementia) were excluded. Additionally, one reviewed study (Weintraub et al., 2000) and several more identified during the study selection process failed to state whether their sample's KS was alcohol-related or caused by some other etiology (e.g., bariatric surgery), requiring reviewers to contact the authors directly. The distinction between etiologies is important as the two may result in different cognitive profiles (Nikolakaros et al., 2016). Similarly, the only two studies which assessed participants with a specific diagnosis of ARBD provided little participant description, did not refer to diagnostic criteria, and one failed to state whether the sample was inclusive of those with KS or ARD diagnoses (i.e., Horton et al., 2015b).

**Table 11:** Summary of quality assessment outcomes.

	**Outcome**			
**Question**	**Yes (%)**	**No (%)**	**Can't tell (%)**	**Not relevant (%)**
Abstinence achieved?	12 (27.9)	8 (18.6)	23 (53.5)	0 (0)
Diagnostic criteria used?	25 (58.1)	0 (0)	18 (41.9)	0 (0)
Reference standard used?	32 (74.4)	0 (0)	11 (25.6)	0 (0)
Confounding disorders excluded?	26 (60.5)	2 (4.7)	15 (34.9)	0 (0)
Language & culture specific?	41 (95.3)	0 (0)	2 (4.7)	0 (0)
Blinding used?	1 (2.3)	9 (20.9)	25 (58.1)	8 (18.6)
Demographics matched?	22 (51.2)	15 (34.9)	0 (0)	6 (14)
Premorbid ability matched?	31 (72.1)	4 (9.3)	3 (7)	5 (11.6)
Effect sizes reported?	7 (16.3)	32 (74.4)	4 (9.3)	0 (0)
Type-I error minimized?	11 (25.6)	22 (51.2)	0 (0)	10 (23.3)

*Abstinence achieved = ARCI participants at least 6 weeks abstinent; Diagnostic criteria used = samples diagnosed according to accepted diagnostic criteria for ARCI; Reference standard used = ARCI diagnoses confirmed using a combination of accepted methods; Confounding disorders excluded = participants with confounding disorders (e.g., dementia) excluded; Language & culture specific = tools used suitable to population tested; Blinding used = administrators blind to diagnoses when making between group comparisons; Demographics matched = participants matched for gender and age or differences accounted for in analyses; Premorbid ability matched = participants matched for education and/or premorbid ability or differences accounted for; Effect sizes reported = effect sizes reported where relevant; Type-I error minimized = risk of type 1 error considered and accounted for when making multiple between group comparisons*.

Methodological and statistical errors were also frequent. Firstly, there was a risk of circularity in studies exploring the sensitivity of tests as many authors reported using neuropsychological assessment to inform diagnostic decisions, thereby inflating the apparent diagnostic value of the test observed on repeat testing. This was noted by Wester et al. (2013c) as a particular issue in their evaluation of the RBMT-3's sensitivity to KS, though was avoided by Wester et al. (2013b) in their appraisal of the MoCA in KS and ALC participants. Secondly, while reviewer calculated effect sizes were typically large between ARCI and control samples, many studies included small sample sizes (72% investigated <25 ARCI participants) suggesting they may have lacked the statistical power to detect more subtle between-group differences (type-II error) or produced misleading statistically significant results (type-I error; Button et al., 2013). Thirdly, most studies failed to report effect sizes when conducting appropriate analyses. While this can often be easily calculated by readers (as done here), multiple authors in the included studies commended the discriminatory value of a test based solely on statistical significance between groups (e.g., Oscar-Berman et al., 2004), which does not provide information regarding the relative size of the discrepancy. Given the additional information provided by their inclusion and the limitations of *P* values (see Sullivan and Feinn, 2012), we recommend all future studies in this field report effect sizes where relevant.

## Discussion

The present review aimed to synthesize and discuss studies evaluating neuropsychological tools used in the assessment of Alcohol-Related Cognitive Impairment (ARCI). Overall, the reviewed studies present a clear picture of widespread cognitive impairment increasing in severity from AL through ALC and on to varying forms of more severe ARCI, highlighting the importance of neuropsychological evaluation. Compared with Horton et al.'s (2015a) previous review on this topic, we reviewed 26 additional studies and discussed multiple outcomes of interest not identified within their review. Findings suggest several tests may be useful in this domain, although—consistent with the conclusions of Horton et al.—most have received little evaluation of their psychometric properties. A summary and discussion of key findings is now presented.

### Cognitive Screening

CSIs are useful in providing a preliminary indication of the severity of cognitive impairment. Both the MoCA and MMSE have demonstrated some value in distinguishing those with ARCI from those without and between gradations of alcohol-related impairment, though the MoCA appears the superior of the two (Wester et al., 2013c; Oudman et al., 2014). Although, clinicians using CSIs with this population should be cognizant of the considerable risk of false-positive and false-negative outcomes and avoid making definitive diagnostic decisions purely on the basis of their use.

In the wider literature, additional support for the MoCA comes from Pelletier et al. (2016) who found it proved useful in monitoring improvements in cognitive function in ALs undergoing rehabilitation. However, it is not clear whether the authors' findings simply reflect a learning effect, given the short duration *(M* = 33.5 days, *SD* = 7) between the two administration points. CSIs have also been criticized in the wider literature for their poor sensitivity to alcohol-related memory disorders (Benedict and Brandt, 1992; Munro et al., 2001). Consequently, additional memory testing may be useful during initial assessments. Additionally, CSIs may be of little use in discriminating between ARCI and other neurocognitive disorders such as Alzheimer's, with little observable difference in MMSE scores between groups (Osuntokun et al., 1994; Saxton et al., 2000). Overall, while CSIs provide quick, easily administered assessments of cognitive function, there is a need for more comprehensive testing following screening to ensure correct diagnosis.

### Memory Assessment

#### Episodic Memory

As expected, ARCI participants in the included studies consistently displayed severe deficits on episodic memory tests. Overall, those with KS appear to perform poorly on both recall and recognition variables (Wester et al., 2013a,b, 2014). Those with non-KS ARCI and ALs, however, display deficits mostly on free recall trials, with relatively preserved recognition performance (Saxton et al., 2000; Munro et al., 2001; Wester et al., 2013b). The memory deficit of ALs can often be distinguished from that of KS not only by lesser severity, but also by a faster rate of learning which is comparable to the rate of healthy individuals (Brokate et al., 2003).

Both the RBMT-3 and CVLT discriminate well between KS, ALC and non-ALs, with an overall graded picture of increasing memory impairment from AL through ALC to KS on both measures. Compared with the RBMT-3, the CVLT appears slightly more sensitive to ARCI and provides a more comprehensive overview of verbal memory and learning indices. Still, the RBMT-3 demonstrates comparably excellent sensitivity and specificity and may provide clinically useful information regarding the implications of the memory deficit for a person's day-to-day functioning. The WMS is also sensitive to the memory deficits of those with ARCI, testing both episodic and working memory for verbal and visual stimuli. The WMS has, however, been criticized for its long administration time and cumbersome scoring system, which may preclude use in clinical settings (Kent, 2013). Yet, more accurate assessments of memory function depend on detailed testing, for which the WMS may be useful. Validation of the latest version of the test, the WMS-IV, is required for this group.

#### Autobiographical Memory

Individuals with KS display temporally graded results on autobiographical memory tests, with more recent memories being most impaired, memories from early adulthood often best preserved and childhood memories slightly impaired in comparison (although these last two periods can vary in which is best remembered; Kopelman, 1989; Rensen et al., 2016). The decline in preserved memories from early adulthood to more recent memories in KS is particularly steep, significantly more so than the decline observed over the same period in those with Alzheimer's disease (Kopelman, 1989). The AMI appears superior to the AI, demonstrating superior inter-rater reliability and greater discriminatory ability between KS and control groups (Rensen et al., 2016).

#### Procedural Memory

Mixed findings have been observed in relation to procedural memory function in those with ARCI, likely because the cognitive sequelae of chronic alcoholism—including episodic memory deficits, executive dysfunction, and visuospatial problems—make the isolated assessment of procedural learning challenging in this population (Hayes et al., 2012). For example, the procedural aspect of Tower-Tests may only be accessible after the initial problem-solving element is addressed. Wester et al. (2013a) have suggested the RBMT-3's new Novel Task may assess procedural learning, though probably also recruits episodic memory and visuospatial processes for completion. Nonetheless, given the ease of administering it within the RBMT-3 battery, the Novel Task may provide a valuable addition to clinical memory assessment in those with ARCI. The discussion of procedural memory assessment presented here is circumscribed by our decision to focus on standardized, normatively defined neuropsychological tests (many investigations of procedural memory in those with ARCI rely on experimental procedures). For a comprehensive overview of this domain, we recommend the reviews of implicit memory function and procedural learning in KS provided by Hayes et al. (2012) and Oudman et al. (2015), respectively.

#### Working Memory

Individuals with all forms of ARCI display impairments in working memory (van Geldorp et al., 2012), as do ALs to a lesser extent (Pitel et al., 2008). The BP-Task appears sensitive to the working memory deficits associated with alcoholism, particularly at longer delay times (Ryan and Butters, 1980; Leng and Parkin, 1989), though is also dependent on an ability to shift attention away from the distracting activity and not perseverate. Tasks relying on immediate recall of verbal or visual stimuli may not reveal deficits in working memory (Blackburn and Tyrer, 1985; van Geldorp et al., 2012), with some delay necessary for identifying deficits. Psychometric validation of tests specifically designed to test working memory in those with ARCI is scant and therefore warranted.

#### Confabulation

The early stage of WKS is associated with both provoked and spontaneous confabulations. Accordingly, the assessment of confabulatory behavior may provide important diagnostic information (Borsutzky et al., 2008; Rensen et al., 2017). The use of the Intrusions variable of the CVLT as an index of confabulation is questionable. The findings from Rensen, Oosterman, van Damme, Griekspoor, Wester and Kopelman (2015, 2017) suggest intrusions, while related to confabulations, may be distinct phenomena. The NVCL-20 observational scale for measuring confabulatory behavior appears more suitable for assessing confabulation in those with ARCI, demonstrating superior psychometric properties to other measures used for this purpose. The NVCL-20 is the only confabulation measure to be specifically validated in an ARCI population and to provide a quantification of spontaneous confabulation.

### Executive Function

The testing of EF represents an important element of ARCI assessment, particularly as impairments may have deleterious consequences for rehabilitation and treatment efforts (Bates et al., 2002, 2005). Executive dysfunction is now an accepted feature of KS, with impaired performance in tasks requiring planning (Joyce and Robbins, 1991), rule detection and following (Kopelman, 1991), verbal fluency (Fujiwara et al., 2008), and cognitive estimation (Taylor and O'Carroll, 1995). Compared with ALs, KS demonstrate considerably poorer performance on tests of EF (Maharasingam et al., 2013), leading some to conclude that such impairments represent unique sequelae of organic KS and are not the result of alcohol neurotoxicity (Krabbendam et al., 2000; Brokate et al., 2003). Nonetheless, the literature is replete with evidence of impaired performance on various measures of EF in ALs compared with controls (Moriyama et al., 2002; Oscar-Berman et al., 2004; Pitel et al., 2007), which does not appear to be accounted for by differences in age or education. Thus, it seems the severe executive deficits of KS are superimposed on top of existing, albeit milder, impairments resulting from chronic alcoholism and related factors; although this could include sub-clinical episodes of thiamine deficiency (Pitel et al., 2011). Findings relating to samples with diagnoses other than KS are scant, though one study found 65% of a small sample (*n* = 16) with ARBD performed within impaired ranges on a test of EF (Horton et al., 2015b), but it is not clear from the sample description whether this included individuals with WKS diagnoses.

Despite substantially more impaired executive abilities observed in those with KS compared with ALs, less sensitive measures of EF such as the MoCA's EF subtest may result in little difference between these groups (Wester et al., 2013c), emphasizing the need for more sensitive measures. The ecologically-based battery assessment of EF, the BADS, appears sensitive to KS-related deficits across multiple subtests and can differentiate these from AL impairments (Maharasingam et al., 2013). KS performance on the BADS is not consistent across subtests, highlighting the need to examine subtest scores in addition to the overall profile score (Van Den Berg et al., 2009). In terms of ecological validity, high scores on the Profile score and Temporal Judgment and Zoo Map sub-tests have been associated with subsequent occupational success in ALs, though no link between BADS scores and drinking outcomes was observed (Moriyama et al., 2002).

Individuals with KS perform particularly poorly on tests of rule derivation such as the WCST, MCST, and BPSAT compared with controls and those with psychiatric disorders and other neurological conditions (Shoqeirat et al., 1990; Van Den Berg et al., 2009). Multiple studies have also found evidence of impaired cognitive estimation in KS (Kopelman, 1991; Taylor and O'Carroll, 1995), although an earlier study found preserved performance (Leng and Parkin, 1988). The commonly used test of cognitive estimation, the CET, has previously been criticized for being culturally bound, but this issue has recently been resolved with the production of a revised CET (MacPherson et al., 2014). Accurate cognitive estimations, however, may rely on the retrieval of knowledge from semantic memory and the subsequent error checking of this process as well as executive abilities (Taylor and O'Carroll, 1995). This view has since been corroborated in KS participants using an alternative test of cognitive estimation (Brand et al., 2003) and supported in a review of findings from various clinical groups (Wagner et al., 2011).

There appears to be little inter-correlation between tests of single EFs, including the CET, FAS, WCST, and MCST (Shoqeirat et al., 1990; Kopelman, 1991). Dissattenuated correlations (i.e., correlations corrected for the unreliability of the test scores) between the EF test scores were, however, not considered here and may be stronger than those originally reported (Schmidt and Hunter, 1996). Nonetheless, the lack of correlations is perhaps unsurprising as, although estimations of quantities, verbal fluency, and rule detection may depend on some common skills (e.g., attention), they likely recruit different skills for their completion. This view is consistent with studies using latent variable analyses to study the diversity of executive abilities which have concluded that EFs are separable but related functions that share some level of underlying commonality (e.g., Miyake et al., 2000; Friedman et al., 2008). The resulting models of EF from these investigations distinguish between key functions such as shifting, inhibition and updating, with some also including working memory and planning (Snyder et al., 2015). Such models could provide a useful theoretical basis for testing EFs in those with ARCI; although the EF tests identified here mostly assess shifting (e.g., WCST, BSPAT) and planning (e.g., BADS Zoo Map Test), with tests of fluency and cognitive estimations not neatly fitting within existing models of EF (see Snyder et al., 2015). Inhibition can be tested using the BADS or CANTAB response inhibition tests reviewed here, while updating is typically tested by paradigms such as the Spatial *2*-back or Keep Track tests used by Friedman et al. (2008), indicating a need to incorporate standardized updating tasks into existing batteries.

### Intelligence and Test Batteries

Impairments on intelligence tests have also been observed in those with ARCI, particularly on subtests that involve processing speed, working and episodic memory, and executive abilities. Only two test batteries were evaluated by included studies, the WAIS and CANTAB. The WAIS is often viewed as the gold-standard assessment of intelligence and general ability (Hayes et al., 2016). Those with ARCI, particularly KS, demonstrate better performance on the Verbal IQ (VIQ) components of the WAIS compared with the Performance IQ (PIQ) components; though the reverse has also been documented (Oscar-Berman et al., 1993), highlighting heterogeneity within this population. A depressed WMS relative to WAIS IQ or VIQ may be indicative of KS, though we do *not* recommend using this discrepancy to diagnose the condition. The process has been repeatedly criticized based on several inherent flaws (Dennis et al., 2009; Kent, 2013). First, inferring memory dysfunction from the discrepancy is fallible, as an accurate measure of premorbid function from which any deterioration in IQ can also be established is rarely available. Second, those at extreme ends of the IQ spectrum may produce misleading discrepancy scores. Third, WMS and WAIS performance are not exclusive as there is overlap between the skills used in both, particularly working memory processes (Shipstead et al., 2016). Further, the historical view of KS as a circumscribed memory disorder is clearly challenged by the extant evidence, with wide-ranging cognitive deficits observed in the studies reviewed here. Indeed, Bowden (1990) has argued that the strict selection criteria used for KS by some researchers may have resulted in samples which were unrepresentative of the more varied presentation typical of this population. Accordingly, Scalzo et al. (2015) report a case-series investigation of those with WKS whose IQ minus MQ scores ranged widely from−14 to 36, demonstrating the poor diagnostic validity of the discrepancy approach.

Tests from the computerized CANTAB battery appear sensitive to the deficits associated with ARCI, namely episodic memory and EF (Horton et al., 2015b). The battery is psychometrically and practically appealing as its on-screen nature allows little room for administration or interpretation errors, parallel versions of tests are available, and normed scores are calculated using a large and diverse normative database. The tests are also almost entirely visual in style and are therefore unaffected by low verbal capabilities or cultural background.

Overall, intelligence and battery assessments appear useful for evaluating multiple cognitive abilities while using the same normative sample for comparison. In addition, composite scores derived from battery tests are typically more reliable and better predictors of functional outcomes than single test scores (Harvey, 2012). However, large batteries such as the WAIS can be time-consuming and demanding for some, potentially explaining why many studies have used only a selection of relevant subtests as opposed to the entire battery. Additionally, most batteries will require the inclusion of auxiliary tests to cover all functions known to be impaired in those with ARCI, such as orientation or aspects of memory and learning not assessed.

### Premorbid Ability

The assessment of premorbid intellectual function in those with ARCI is essential for correctly interpreting the extent of the suspected cognitive deficit. The NART may provide some value in predicting premorbid function in those with ARCI and the revised version appears more effective than the original. Still, it remains unknown whether NART performance is entirely impervious to the deficits associated with KS. Nonetheless, the measure appears to be the common choice with this population and the NART-R has recently been re-standardized in a non-clinical British sample for the new WAIS-IV (Bright et al., 2016), increasing its application.

### Further Considerations in the Neuropsychological Assessment of ARCI

In addition to test selection, several additional choices face clinicians using neuropsychological measures to assess ARCI. One of the foremost of these is the decision as to when tests should be administered. A systematic review by Walvoort et al. (2013) found it typically requires 6-weeks of abstinence before reliable neuropsychological performance can be achieved. However, the use of cognitive screening tests during or immediately following detoxification is common practice and may provide some indication of a person's cognitive ability (Royal College of Psychiatrists, 2014); although results should be interpreted in the light of Walvoort and colleagues' findings. The use of repeat testing may also be important to track the changes in cognition that have been observed during the first 2 years post-detoxification (Stavro et al., 2013). The Royal College of Psychiatrists (2014) in the UK have suggested that following initial assessments, more detailed cognitive assessments should be undertaken after 3-months, then repeated at 6-month intervals for 3 years. This emphasizes the need to use tests that have high test-retest reliability values and/or parallel versions to avoid practice effects.

Several person-specific factors should also be considered when interpreting the outcomes of cognitive testing in those suspected of ARCI to ensure accurate interpretation. First among these is the consideration of any comorbid psychopathology, as individuals with conditions such as depression (Bosaipo et al., 2017) and schizophrenia (Bora and Pantelis, 2015) have been found to display decreased performance on various cognitive tests compared with matched controls. Relatedly, psychiatric medications can have varying effects on cognitive performance, both during and immediately following use (e.g., Bouchard et al., 2015; Helmes and Ostbye, 2015). Additional non-medical substance use including cocaine, heroin, cannabis, and even (acutely) nicotine can also have deleterious effects on cognitive testing lasting for varying periods following abstinence, particularly in those with co-morbid psychiatric disorders (see Gould, 2010); though one study has found no additional impact of cannabis use on MoCA scores in ALs (Alarcon et al., 2015), suggesting the effects may vary. Finally, the presence of cerebrovascular disease and traumatic brain injuries should also be considered due to their high prevalence (25%) within this population (Wilson et al., 2012). Together, these findings highlight the need to combine neuropsychological testing with a clinical diagnostic process and other assessment measures—primarily neuroimaging and nutritional assessment (Scalzo et al., 2015)—to ensure accurate diagnosis.

### Diagnostic Distinctions

As mentioned, few studies included in the review investigated non-KS forms of ARCI, suggesting further investigations of those with other diagnostic labels (e.g., ARD, ARBD) may be warranted. However, the concept of a prolonged alcohol-related neurocognitive disorder with a distinct neuropathological basis from WKS or other disorders of vitamin depletion (e.g., Pellagra) remains tenuous. Given the similarities in cognitive profiles between groups with diagnoses of ARD, ARBD and KS seen here, the present review findings appear consistent with suggestions that common neuropathological processes, namely thiamine depletion, underpin most forms of ARCI (Joyce, 1994; Zahr et al., 2011). This theory is supported by research with an AL population demonstrating a significant correlation between thiamine levels and memory performance, as well as a graded decrease in cognitive ability with each symptom of WE (Caine et al., 1997 criteria) present (Pitel et al., 2011).

Similar cognitive profiles are also evident across gradations of alcohol-related impairment, suggesting less impaired groups sit earlier in the same underlying pathological process as those with KS. Indeed, AL & ALC groups appear to be impaired, to a lesser extent, on many of the same tests of memory (e.g., Pitel et al., 2008; Wester et al., 2013b) and general cognitive ability (e.g., Wester et al., 2013c) as those with KS. However, those with KS typically display impaired performance on tests of recognition memory and orientation, yet ALC and ARD populations perform at control-level (Munro et al., 2001; Wester et al., 2013b), indicating that some neuropsychological distinctions (other than those of degree) are evident when thiamine depletion progresses to the point of a KS diagnosis. Nonetheless, normal recognition memory and an absence of disorientation have been observed in a case of KS meeting standard diagnostic criteria for the disorder (Noël et al., 2001), further highlighting the heterogeneity within this population.

The present findings suggest it may be valuable from a clinical perspective to adopt a broad diagnostic conceptualization of ARCI, assuming a degree of individual variability in the particular functions impaired and where the extent of impairment is the deciding factor in diagnostic decision making; as opposed to trying to decide into which of the highly overlapping diagnostic categories (e.g., ARD, WKS) an individual best fits. Those adopting this view could acknowledge the central role of thiamine deficiency in producing ARCI (Arts et al., 2017), and still specify when a person's symptomology closely resembles KS, whilst also recognizing that various other etiological factors (e.g., other nutritional deficiencies, impaired liver function etc.) can, like thiamine depletion, contribute to varying degrees and may explain heterogeneity in degree and types of impairment observed (see Bowden, 1990). The more transient, but still significant (e.g., Stavro et al., 2013), effects of alcohol neurotoxicity (Arts et al., 2017) should also be recognized within this broad approach, highlighting to clinicians the potential for recovery of certain functions over time. This perspective closely resembles the DSM-5's alcohol-related neurocognitive disorder, which bifurcates twice into mild and severe forms and from the latter into amnestic and non-amnestic types. The ARBD conceptualization postulated by Wilson et al. (2012)—which encompasses WKS and all clinically significant forms of ARCI not meeting the criteria for WKS—may also provide a highly pragmatic diagnostic conceptualization for clinical use consistent with the above approach. That said, if as the literature appears to suggest, that most cases of ARCI are variants of WKS caused by thiamine deficiency to a greater or lesser extent, then the term WKS may be more apt from an etiological perspective and has recently been advocated for use in place of terms like ARD or ARBD (Arts et al., 2017). Though, as mentioned, traditional views of WKS may fail to fully capture the syndrome as experienced by alcohol users in regards to its heterogeneity and the contributing role of etiological factors other than thiamine depletion, particularly alcohol neurotoxicity. Nonetheless, the value of neuropsychological assessment lies in characterizing the type and degree of cognitive dysfunction suffered by alcohol-users and monitoring it over time, regardless of diagnostic categories and nomenclature.

### Directions for Future Research

Overall, we recommend further appraisal of all tests discussed here in those with ARCI, but a selection of more specific recommendations for future studies are worthy of mention. First, the generalizability of findings and methodological rigor of future research in this area can be improved by addressing some of the limitations of the existing literature identified here, including clear reporting of abstinence duration, diagnostic criteria (particularly if assessing samples with “ARBD”), and the methods used for diagnosis (including whether the tests evaluated contributed to diagnostic decisions). Second, further evaluations of the test re-test reliability of tests used with this population are necessary to ensure any variability in scores on repeat testing represent true differences, not practice effects. Third, to support clinicians in identifying those with or at risk of ARCI, it would be beneficial for studies to continue to identify test cut-off scores which delineate gradations of impairment (e.g., from AL to ALC and more severe forms of ARCI), as has recently been done for the MoCA and MMSE (Wester et al., 2013c; Oudman et al., 2014). Fourth, it would also be useful to see how well performance on tests reflects the ability to carry out activities of daily living and can predict functional outcomes such as treatment adherence. While excluding treatment studies from the present review decreased the chance of identifying these outcomes, a brief review of the ARCI treatment and intervention literature did not find any such investigations.

Fifth, many of the most commonly employed neuro-psychological tools identified in Table 1 were not evaluated by any studies in the review and require investigation to support their continued use with this population. Two additional tests requiring validation in this population are the Addenbrooke's Cognitive Examination-III (ACE; Hsieh et al., 2013) and Repeatable Battery for the Assessment of Neuropsychological Status (R-BANS). In an on-going investigation of ARCI prevalence by the present authors, these are the most commonly reported neuropsychological tools used by UK clinicians in the ARCI diagnostic process. The ACE has demonstrated screening abilities commensurate with the MoCA and superior to the MMSE for AL impairments (Ridley et al., 2017), but neither the ACE nor R-BANS have been evaluated for use in ARCI diagnosis.

Finally, two CSIs have recently been developed specifically for assessing alcohol-related cognitive impairments, the Brief Examination of Alcohol-Related Neuropsychological Impairments (BEARNI; Ritz et al., 2015) and the Test of Detection of Cognitive Impairment in Alcoholism (TEDCA; Jurado-Barba et al., 2017), though both have only been validated in AL populations not meeting ARCI diagnostic criteria (both CSIs can be accessed in the corresponding publications [the BEARNI is in French]). The BEARNI was designed as an easily administered CSI for assessing the deficits associated with alcoholism: working and episodic memory, visuospatial abilities, EF, and ataxia. In its validation, Ritz et al. (2015) found the BEARNI was considerably more sensitive when differentiating between ALs and controls (sensitivity = 98.4%) compared with the MMSE (9.5%) and Dementia Rating Scale (7.9%; Coblentz et al., 1973). However, the tool displayed relatively poor specificity (50%). The TEDCA appears highly similar to the BEARNI in its assessment of working and episodic memory and visuospatial skills. The TEDCA's assessment of EF, however, appears more comprehensive, including the Trail Making Test-B, conceptual similarity tests (e.g., “What do food & gasoline have in common?”), and a go-nogo task; compared with only a category fluency test in the BEARNI. The TEDCA demonstrated moderate sensitivity (67%) and good specificity (76.7%) when differentiating between ALs and controls (Jurado-Barba et al., 2017), although these findings are obfuscated by the failure to state whether ALs were abstinent during testing. Overall, both tests are valuable additions to the assessment of this population and possess several strengths: short administration time, an ability to be administered by non-specialists, and a focus on the key areas of alcohol-related cognitive impairment. A priority for future research in this area should be to evaluate the screening capabilities of both tests for those with ARCI diagnoses such as KS.

### Limitations of the Review

The present review may be limited by the exclusion of studies evaluating non-standardized tests without normative data, as there may be tests not meeting these criteria which are particularly useful in the assessment of ARCI. However, expanding the review to include such tests would be beyond the scope of a single review and the evaluation of standardized normatively defined tests is likely of most value to those involved in the assessment of ARCI. Second, this review considered only one test of affect or social cognition (WMS-III Face Recognition Test; Holdnack and Delis, 2004) indicating the lack of studies evaluating standardized tests of these domains. If considering ARCI from a dual-processing perspective (e.g., Helfrich et al., 2018), as has recently been advocated (Brion et al., 2015), then the tests reviewed here focus mostly on the “reflective system” system (which draws upon memory and EF to initiate considered, deliberate responses), with few assessing the affective component of the “affective-automatic” system responsible for the impulsive processing of stimuli, including decoding the affective quality of a stimulus. This is notable as tests of affect and social cognition may prove informative for those involved in the treatment of this population (Brion et al., 2015). For example, ALs and those with KS display impaired processing of emotional facial expressions and, in the former group, this has been associated with interpersonal dysfunction (Kornreich et al., 2001; Montagne et al., 2006; Donadon and Osório, 2014). Thus, tests of affect should also be considered when assessing ARCI; to date, however, studies in this domain are scant (Arts et al., 2017).

Third, we did not include studies evaluating neuropsychological tools in AL participants, which could have provided additional information on the utility of tests for the assessment of alcohol-related deficits. However, we have introduced some of the more pertinent and up-to-date evidence relating to ALs (e.g., Alarcon et al., 2015; Ritz et al., 2015) into our discussion to supplement the review findings. Fourth, several studies in the review evaluated older versions of tests used today (e.g., WMS/WAIS editions), thus caution should be taken in applying these findings to newer versions. Although older versions are likely conceptually and structurally similar, they should not be viewed as the same test (Strauss et al., 2006). Finally, the findings from older studies in the review should be interpreted carefully as they were considerably more likely than newer studies to contain methodological weakness (e.g., lack of diagnostic criteria and short abstinence duration).

## Conclusions and Recommendations

Through an extensive review of the literature in this area, the present systematic review has provided a novel overview of ARCI assessment, including a direct understanding of each test's utility compared with other tests of the same function. While evidence concerning the psychometric, diagnostic and practical values of tests in assessing ARCI appears to be in its infancy compared with other disorders (e.g., dementias), the systematic collation and comparison of studies conducted here makes it possible to proffer a number of provisional recommendations for tests in each domain of assessment.

The MoCA is the most well-evidenced CSI of choice in this population, but the ACE, BEARNI, and TEDCA all require validation and comparison to determine the more effective measure. The CVLT and RBMT are recommended for more detailed memory assessment. The WMS is also likely to be of value, and the optional tests of orientation (i.e., WMS-IV Cognitive Status Exam) should be included; although the most recent WMS-IV has not been evaluated in this population. The NVCL-20 should be the choice assessment of confabulation. Rule derivation tests (e.g., WCST) are sensitive measures of executive dysfunction in those with ARCI, with the BSPAT possessing greatest psychometric support. However, the BADS is recommended as a more comprehensive assessment of EF given the differential levels of impairment observed across EF tests in this group. The WAIS is the most commonly used and well-validated measure of intelligence in those with ARCI and its continued use appears justified; particularly if using its memory-focused analog (WMS) to permit direct comparison across functions using the same normative reference data. However, other test batteries (e.g., R-BANS) have received little evaluation of their utility to date. When determining premorbid function, the NART-R is the test of choice, though requires further evaluation in those with KS. Overall, while test selection will ultimately depend on a variety of factors, including the preferences and goals of the administrator, the present findings can assist clinicians and researchers assessing ARCI by supporting efficient, evidence-based decisions.

## Author Contributions

All authors contributed to the conception and design of the review. RH was involved in all stages of the review process and wrote the original manuscript. GR-D contributed to the screening of studies, the interpretation and synthesis of findings, and the revision of the manuscript. BJ contributed to the extraction of data, the appraisal of study quality, the interpretation and synthesis of findings, and the revision of the manuscript. All authors approved the final manuscript before submission.

### Conflict of Interest Statement

The authors declare that the research was conducted in the absence of any commercial or financial relationships that could be construed as a potential conflict of interest.

## Abbreviations

Samples: ALs: Alcohol-dependent persons (no specific diagnosis of cognitive impairment); ALC: Alcohol-dependent persons with a diagnosis of mild alcohol-related cognitive impairment not fitting KS or ARD criteria
ARBD: Alcohol-related brain damage
ARCI: Alcohol-related cognitive impairment
ARD: Alcohol-related dementia
KS: Korsakoff's syndrome
WE, Wernicke's Encephalopathy. Key neuropsychological tests: BADS: Behavioral Assessment of the Dysexecutive Syndrome
CVLT: California Verbal Learning Test
MMSE: Mini-Mental Status Examination
MoCA: Montreal Cognitive Assessment
NART: National Adult Reading Test
NVCL-20: Nijmegen-Venray Confabulation List
RMBT: Rivermead Behavioral Memory Test
WAIS: Wechsler Adult Intelligence Scale
WMS, Wechsler Memory Scale. Other: CSIs: Cognitive Screening Instruments
EF: Executive Function.
