# Supplementary material for: Identification and Evaluation of Neuropsychological Tools Used in the Assessment of Alcohol-Related Cognitive Impairment: A Systematic Review
Source: Front Psychol. 2018 Dec 18;9:2618. doi: 10.3389/fpsyg.2018.02618 (PMC6305333; doi:10.3389/fpsyg.2018.02618)
Supplement: Supplementary file 1 [file Table_1.DOCX]

| **Table 1** Quality assessment checklist | | | | | | |  |
| --- | --- | --- | --- | --- | --- | --- | --- |
|  | | Question | Response | |  |  |  |
| 1. | Was a sufficient period of abstinence (i.e., preferably >2 months but a minimum of >6 weeks) achieved prior to assessment? | | | Yes/ No/ Can’t tell | |  |  |
| 2. | Are participants diagnosed in accordance with (or already diagnosed using) the criteria outlined in nosological systems (DSM or ICD) or other accepted diagnostic criteria (e.g., Oslin & Cary, 2003)? | | | Yes/ No/ Can’t tell | |  |  |
| 3. | Is an ARCI diagnosis confirmed using an appropriate reference standard* prior to or following neuropsychological assessment? | | | Yes/ No/ Can’t tell | |  |  |
| 4. | Were persons with confounding conditions such as traumatic brain injuries or dementia excluded? | | | Yes/ No/ Can’t tell | |  |  |
| 5. | Are the neuropsychological tools used specific to the language and culture of the population tested? | | | Yes/ No/ Can’t tell | |  |  |
| 6. | Are administrators blind to diagnoses where the ARCI diagnosis has been established pre-assessment? | | | Yes/ No/ Can’t tell/ Not appropriate | |  |  |
| 7. | Were participants matched according to demographic variables (age and gender) or were differences accounted for in between group comparisons? | | | Yes/ No/ Can’t tell/ Not appropriate | |  |  |
| 8. | Were participants matched according pre-morbid IQ or were differences accounted for in between group comparisons? | | | Yes/ No/ Can’t tell/ Not appropriate | |  |  |
| 9. | Were effect sizes reported to support analyses where appropriate? | | | Yes/ No | |  |  |
| 10. | Type 1 error potential: did the authors report adjusting the alpha level or consider the risk of type 1 error when multiple statistical comparisons made? | | | Yes/ No/ Can’t tell/ Not appropriate | |  |  |
| All questions answered in relation to the sample/s of focus (i.e., those with/ being tested for ARCI).  *We define a reference standard for ARCI as a multifaceted approach to diagnosis involving at least two of the following: comprehensive neuropsychological assessment, a review of medical history, medical/ physical assessment, neuroimaging investigation.  ^#^Estimations of pre-morbid intelligence may be achieved through specific neuropsychological tests known to be relatively immune to neurological damage (e.g., subscales of the Wechsler Adult Intelligence Scale IV; Wechsler, 2008) or via the collection of relevant demographic information (e.g., years of education; Vakil, 2012). | | | | | | | |

| **Table 2** Quality assessment outcomes | | | | | | | | | | | |
| --- | --- | --- | --- | --- | --- | --- | --- | --- | --- | --- | --- |
| Author(s) | Year | 1. Abstinence | 2. Diagnostic criteria | 3. Reference standard | 4. Confounding disorders excl. | 5. Language & culture | 6. Blinding | 7. Demographics | 8. Pre-morbid ability | 9. Effect sizes | 10. Type-I error |
| 1. Kapur & Butters | 1977 | ⭘ | ⭘ | ● | ● | ● | ⭘ | ⮿ | ● | ⮿ | ⮿ |
| 1. Glosser et al. | 1977 | ⭘ | ⭘ | ⭘ | ⭘ | ● | ⮿ | ⮿ | ⮿ | ⮿ | ⮿ |
| 1. Harbinson | 1984 | ⭘ | ● | ● | ● | ● | ⮿ | ● | ● | ⮿ | ⮿ |
| 1. Butters et al. | 1985 | ⭘ | ⭘ | ⭘ | ⭘ | ● | ⮿ | ⮿ | ⮿ | ⮿ | ⮿ |
| 1. Kopelman | 1986 | ● | ⭘ | ● | ⭘ | ● | ⭘ | ● | ● | ⮿ | ⮿ |
| 1. Mazzuchi et al. | 1987 | ⭘ | ⭘ | ⭘ | ● | ⭘ | ⭘ | ⮿ | ● | ⮿ | ● |
| 1. Alekoumbides et al. | 1987 | ⭘ | ⭘ | ⭘ | ⮿ | ● | ⭘ | ⮿ | ● | ⮿ | ⮿ |
| 1. Charter & Alekoumbides | 1988 | ⭘ | ⭘ | ⭘ | ⮿ | ● |  | ⮿ | ● | ⮿ |  |
| 1. Crawford et al. | 1988 | ⭘ | ● | ● | ● | ● | ⭘ | ● | ● | ⮿ | ⮿ |
| 1. Leng & Parkin | 1989 | ⭘ | ⭘ | ⭘ | ⭘ | ● | ⭘ | ● | ● | ⮿ |  |
| 1. Shoqeirat et al. | 1990 | ⭘ | ⭘ | ⭘ | ⭘ | ● | ⭘ | ⮿ | ● | ⮿ | ⮿ |
| 1. Delis et al. | 1991 | ⭘ | ● | ⭘ | ⭘ | ● | ⭘ | ⮿ | ⮿ | ⮿ | ● |
| 1. Deary et al. | 1991 | ● | ⮿* | ● | ● | ● | ⭘ | ⮿ | ● | ⮿ | ⮿ |
| 1. Kopelman | 1991 | ● | ● | ⭘ | ⭘ | ● | ⮿ | ⮿ | ● | ⮿ | ⮿ |
| 1. O’Carroll et al. | 1992 | ● | ● | ● | ● | ● | ⭘ | ● | ● | ⮿ | ⮿ |
| 1. Oscar-Berman et al. | 1993 | ⮿ | ⭘ | ● | ● | ● | ⭘ | ● | ⮿ | ⮿ | ⮿ |
| 1. Duffy & O’Carrol | 1994 | ● | ● | ● | ● | ● | ⭘ | ● | ● | ⮿ | ⮿ |
| 1. Taylor & O’Carrol | 1995 | ⭘ | ● | ● | ● | ● | ⭘ | ⮿ | ● | ⮿ | ⮿ |
| 1. Welch et al. | 1997 | ⮿ | ⭘ | ● | ⭘ | ● | ⮿ | ⮿ | ● | ⮿ |  |
| 1. Beaunieux et al. | 1998 | ⭘ | ⭘ | ● | ● | ● |  | ● | ● | ⮿ |  |
| 1. Woodburn & Johnstone | 1999 | ⭘ | ● | ● | ● | ● | ⭘ | ⮿ | ⮿ | ⮿ | ● |
| 1. Woodburn & Johnstone | 1999 | ⭘ | ● | ● | ● | ● | ⭘ | ⮿ | ⮿ | ⮿ | ● |
| 1. Weintraub et al. | 2000 | ⭘ | ⭘ | ⭘ | ⭘ | ● | ⭘ | ⮿ | ⮿ | ⮿ | ● |
| 1. Taylor & Heaton | 2001 | ⮿ | ⭘ | ● | ● | ● | ⭘ |  |  | ⮿ |  |
| 1. Bright et al. | 2002 | ⭘ | ⭘ | ● | ● | ● | ⭘ | ● | ● | ⮿ | ⮿ |
| 1. Brokate et al. | 2003 | ⮿ | ● | ● | ⭘ | ● | ⭘ | ⮿ | ● | ⮿ | ⮿ |
| 1. Oscar-Berman et al. | 2004 | ⮿ | ⭘ | ● | ● | ● | ⮿ | ⮿ | ● | ⮿ | ⮿ |
| 1. Holdnack & Delis | 2004 | ⮿ | ⭘ | ● | ⭘ | ● | ⭘ | ● | ● | ⮿ | ● |
| 1. Wester | 2007 | ⭘ | ● | ● | ● | ● |  |  |  |  |  |
| 1. Piekema et al. | 2008 | ⭘ | ● | ● | ⭘ | ● | ⭘ | ● | ● | ⮿ |  |
| 1. Pitel et al. | 2008 | ⮿ | ● | ● | ● | ● | ⭘ | ● | ● | ⮿ | ⮿ |
| 1. Van Den Berg et al. | 2009 | ⭘ | ● | ● | ● | ● | ⭘ | ● | ● | ● | ⮿ |
| 1. Van Oort & Kessels | 2009 | ● | ● | ● | ● | ⭘ |  |  |  |  |  |
| 1. Maharasigngam et al. | 2013 | ⭘ | ● | ● | ● | ● |  | ● | ● | ● | ● |
| 1. Wester et al. | 2013 | ● | ● | ● | ● | ● | ⭘ | ● | ● | ⮿ | ● |
| 1. Wester et al. | 2013 | ● | ● | ● | ● | ● | ⭘ | ● | ● | ● | ● |
| 1. Wester et al. | 2013 | ⮿ | ● | ● | ⭘ | ● | ⮿ | ● | ● | ● | ● |
| 1. Wester et al. | 2014 | ● | ● | ● | ● | ● |  | ● | ● | ● | ● |
| 1. Oudman et al. | 2014 | ● | ● | ● | ● | ● | ⭘ | ● | ● | ⮿ |  |
| 1. Horton et al. | 2015 | ● | ⭘ | ⭘ | ⭘ | ● |  |  |  |  |  |
| 1. Rensen et al. | 2015 | ● | ● | ● | ● | ● | ⮿ | ● | ● | ⮿ | ⮿ |
| 1. Rensen et al. | 2016 | ⭘ | ● | ● | ● | ● | ● | ● | ● | ● | ⮿ |
| 1. Rensen et al. | 2017 | ⭘ | ● | ● | ● | ● |  |  |  |  |  |
| Note: ● = yes ⮿ = no; ⭘ = can’t tell; no circle = not relevant. * = not diagnosed according to standardised criteria provided in nosological systems (e.g., ICD), but the author(s) provide the criteria used for diagnosis and these appear consistent with the contemporary understanding. | | | | | | | | | | | |

| **Table 3** ARCI diagnostic criteria used in reviewed studies (quality assessment checklist, Question 2) | | |
| --- | --- | --- |
| Diagnosis | Criteria | Number of studies |
| Korsakoff’s Syndrome | DSM (III-R – V) | 15 |
|  | Kopelman (2002) | 7 |
|  | ICD (9 – 10) | 2 |
|  | Kopelman, Thomson, Guerrini, and Marshall (2009) | 2 |
|  | Caine, Halliday, Kril, and Harper (1997) | 2 |
|  | Butters and Cermak (1980) | 1 |
|  | Cutting (1978) | 1 |
| Alcohol-Related Dementia | DSM (Dementia, general) | 2 |
|  | DSM (Alcoholic Dementia) | 1 |
| Mild neurocognitive disorder due to alcohol | DSM-V | 4 |
| Alcohol-Related Brain Damage | - | 0 |
| Note: DSM = Diagnostic Statistical Manual; ICD = International Classification of Diseases | | |

**Comment:** references for all 43 reviewed studies can be found in the main article and in Supplemental Document 2: Neuropsychological tests used to assess ARCI

**References**

Butters, N., & Cermak, L. S. (1980). The persistence of amnesia: Data, theory and methods. *Journal Of Clinical Neuropsychology, 2*(4), 343-353. doi:http://dx.doi.org/10.1080/01688638008403805

Caine, D., Halliday, G. M., Kril, J. J., & Harper, C. G. (1997). Operational criteria for the classification of chronic alcoholics: identification of Wernicke's encephalopathy. *Journal Of Neurology, Neurosurgery, And Psychiatry, 62*(1), 51-60. doi:10.1136/jnnp.62.1.51

Cutting, J. (1978). The relationship between Korsakov's syndrome and 'alcoholic dementia'. *British Journal of Psychiatry, 132*(3), 240-251.

Kopelman, M. D. (2002). Disorders of memory. *Brain, 125*(10), 2152-2190. doi:https://doi.org/10.1093/brain/awf229

Kopelman, M. D., Thomson, A. D., Guerrini, I., & Marshall, E. J. (2009). The korsakoff syndrome: Clinical aspects, psychology and treatment. *Alcohol and Alcoholism, 44*(2), 148-154. doi:https://doi.org/10.1093/alcalc/agn118

Oslin, D. W., & Cary, M. S. (2003). Alcohol-related dementia: Validation of diagnostic criteria. *The American Journal of Geriatric Psychiatry, 11*(4), 441-447. doi:http://dx.doi.org/10.1176/appi.ajgp.11.4.441

Vakil, E. (2012). Neuropsychological assessment: Principles, rationale, and challenges. *Journal Of Clinical And Experimental Neuropsychology, 34*(2), 135-150. doi:10.1080/13803395.2011.623121

Wechsler, D. (2008). *Wechsler Adult Intelligence Scale—Fourth Edition*. San Antonio, TX: Pearson.
