# Supplementary material for: Identification and Evaluation of Neuropsychological Tools Used in the Assessment of Alcohol-Related Cognitive Impairment: A Systematic Review
Source: Front Psychol. 2018 Dec 18;9:2618. doi: 10.3389/fpsyg.2018.02618 (PMC6305333; doi:10.3389/fpsyg.2018.02618)
Supplement: Supplementary file 2 [file Table_2.DOCX]

| **Table 1** Standardised neuropsychological tools used in the assessment of ARCI | | | | | | | | |
| --- | --- | --- | --- | --- | --- | --- | --- | --- |
| Cognitive screening | Memory | Executive function | Intelligence and test batteries | Premorbid function | Visual perception | Language | Motor function | Attention |
| Mini-Mental Status Examination (*n* = 23)^#^  Dementia Rating Scale (*n* = 6) ^#^  CAMCOG (*n* = 6) ^#^  Montreal Cognitive Assessment (*n* = 2) ^#^  Addenbrook’s Cognitive Examination (*n* = 1) | Wechsler Memory Scale-I/ II/ III (*n* = 38/ 12/ 4)^#^  Rey-Osterrieth Complex Figure Test (*n* = 21)  Rivermead Behavioural memory Test/ -3 (*n* = 6/ 5)^#^  California Verbal Learning Test (*n* = 10)^#^  Rey-Osterrieth Auditory Verbal Learning Test (*n* = 10)  Warrington Recognition Memory Test (*n* = 7)  Brown-Peterson Task (*n* = 7)^#^  Benton Visual Retention Test (*n* = 6)  Free & Cued Selective Reminding Test (*n* = 3)^#^  Corsi Block Tapping Test *n* = 2) ^#^  Doors & People Test (*n* = 1)^#^  Three Words Three Shapes (*n* = 1) ^#^  Benton Facial Recognition Test (*n* = 1)  Philadelphia (repeatable) Verbal Learning Test (*n* = 1)  Adult Memory & Information Processing Battery (now the Brain Injury Rehabilitation Trust Memory Information Processing Battery; *n* = 1)  Memory for Designs Test (*n* = 1) | FAS Verbal Fluency (*n* = 20)^#^  Stroop Word-Colour Test (*n* = 17)  Wisconsin Card Sorting Test (*n* = 14)^#^  Trial Making Test (*n* = 13)  Modified Card Sorting Test (*n* = 10)^#^  Cognitive Estimation Test/ - Shortened (*n* = 4/ 2)^#^  Behavioural Assessment of the Dysexecutive Syndrome (*n* = 5)^#^  Tower of London / Hanoi (*n* = 3/ 2)^#^  Brixton Spatial Anticipation Test (*n* = 3)^#^  Controlled Oral Word Association Test (*n* = 1)  Ruff Figural Fluency Test (*n* = 1)  Porteus Mazes (*n* = 1) | Wechsler Adult Intelligence Scale-I/ II/ III (*n* = 41/ 30/ 11)^#^  Raven’s Progressive Matrices (*n* = 4)  Consortium to Establish a Registry for Alzheimer’s Disease (*n* = 4)  Leistungs-prüf-system (German; *n* = 4)  Halstead-Reitan Battery (*n* = 3)  Repeatable Battery for the Assessment of Neuropsychological Status (*n* = 1)  Wide-Range Achievement Test (*n* = 1)  CANTAB (*n* = 1)  Neurocognitive Status Examination (*n* = 1)  Hayling Test (*n* = 1)  Culture-Fair Intelligence Test (*n* = 1) | National Adult Reading Test/ -R (*n* = 41/ 2)^#^  Mehrfacjwajhi-Wortschaz Test (German; *n* = 3)  Wechsler Adult Intelligence Scale - Vocabulary Test (*n* = 2)^#^  Wechsler Test of Adult Reading (*n* = 1) | Clock Drawing Test (*n* = 4)  Embedded Figures Test (*n* = 2)  Visual Objects Space Perceptual Battery (*n* = 1) | Boston Naming Test (*n* = 6)  Token Test (*n* = 1)  Graded Naming Test (*n* = 1) | Tactual performance Test (*n* = 1)  Grooved Pegboard (*n* = 1) | D2 Test of Attention (*n* = 1) |
| Note: ^#^ = test evaluated by studies within the narrative synthesis. | | | | | | | | |

**Comment:** The references below are divided into two sections: one including the 43 studies included in the narrative synthesis and the second presenting the 63 additional studies also extracted at phase one.

**References (43 studies included in narrative synthesis)**

Alekoumbides, A., Charter, R. A., Adkins, T. G., & Seacat, G. F. (1987). The diagnosis of brain damage by the WAIS, WMS, and Reitan Battery utilizing standardized scores corrected for age and education. *International Journal of Clinical Neuropsychology, 9*(1), 11-28.

Beaunieux, H., Desgranges, B., Lalevée, C., de la Sayette, V., Lechevalier, B., & Eustache, F. (1998). Preservation of cognitive procedural memory in a case of Korsakoff's syndrome: methodological and theoretical insights. *Perceptual And Motor Skills, 86*(3 Pt 2), 1267-1287, doi: 10.2466/pms.1998.86.3c.1267

Bright, P., Jaldow, E., & Kopelman, M. D. (2002). The National Adult Reading Test as a measure of premorbid intelligence: a comparison with estimates derived from demographic variables. *Journal Of The International Neuropsychological Society: JINS, 8*(6), 847-854, doi:https://doi.org/10.1017/S1355617702860131.

Brokate, B., Eling, P., Hildebrandt, H., Fichtner, H., Runge, K., & Timm, C. (2003). Frontal lobe dysfunctions in Korsakoff's syndrome and chronic alcoholism: Continuity or discontinuity? *Neuropsychology, 17*(3), 420-428, doi:10.1037/0894-4105.17.3.420.

Butters, N., Wolfe, J., Martone, M., Granholm, E., & Cermak, L. S. (1985). Memory disorders associated with huntington's disease: Verbal recall, verbal recognition and procedural memory. *Neuropsychologia, 23*(6), 729-743. doi:http://dx.doi.org/10.1016/0028-3932(85)90080-6

Charter, R. A., & Alekoumbides, A. (1988). An abbreviated version of a psychometric battery for the diagnosis of brain damage utilizing standardized scores corrected for age and education. *International Journal of Clinical Neuropsychology, 10*(3), 123-129.

Crawford, J. R., Parker, D. M., & Besson, J. A. (1988). Estimation of premorbid intelligence in organic conditions. *The British Journal of Psychiatry, 153*, 178-181. doi:http://dx.doi.org/10.1192/bjp.153.2.178

Deary, I. J., Hunter, R., Langan, S. J., & Goodwin, G. M. (1991). Inspection time, psychometric intelligence and clinical estimates of cognitive ability in pre-senile Alzheimer's disease and Korsakoff's psychosis. *Brain, 114*(6), 2543-2554, doi:https://doi.org/10.1093/brain/114.6.2543.

Delis, D. C., Massman, P. J., Butters, N., Salmon, D. P., Cermak, L. S., & Kramer, J. H. (1991). Profiles of demented and amnesic patients on the California Verbal Learning Test: Implications for the assessment of memory disorders. *Psychological Assessment: A Journal of Consulting and Clinical Psychology, 3*(1), 19-26. doi:http://dx.doi.org/10.1037/1040-3590.3.1.19

Duffy, L., & O'Carroll, R. (1994). Memory impairment in schizophrenia: A comparison with that observed in the Alcoholic Korsakoff Syndrome. *Psychological Medicine, 24*(1), 155-165. doi:http://dx.doi.org/10.1017/S0033291700026921

Glosser, G., Butters, N., & Kaplan, E. (1977). Visuoperceptual processes in brain damaged patients on the digit symbol substitution test. *International Journal of Neuroscience, 7*(2), 59-66, doi:http://dx.doi.org/10.3109/00207457709147202.

Harbinson, H. J. (1984). Alcoholic Korsakoff's psychosis: a psychometric, neuroradiological and neurophysiological investigation of nine cases. *The Ulster medical journal, 53*(2), 103-110.

Holdnack, J. A., & Delis, D. C. (2004). Parsing the recognition memory components of the WMS-III face memory subtest: Normative data and clinical findings in dementia groups. *Journal Of Clinical And Experimental Neuropsychology, 26*(4), 459-483.

Horton, L., Duffy, T., & Martin, C. (2015). Neurocognitive, psychosocial and functional status of individuals with alcohol-related brain damage (ARBD) on admission to specialist residential care. *Drugs: Education, Prevention and Policy, 22*(5), 416-427. doi:10.3109/09687637.2015.1050997

Kapur, N., & Butters, N. (1977). Visuoperceptive deficits in long-term alcoholics and alcoholics with Korsakoff's psychosis. *Journal Of Studies On Alcohol, 38*(11), 2025-2035, doi:https://doi.org/10.15288/jsa.1977.38.2025.

Kopelman, M. D. (1986). Clinical tests of memory. *The British Journal of Psychiatry, 148*, 517-525, doi:http://dx.doi.org/10.1192/bjp.148.5.517.

Kopelman, M. D. (1991). Frontal dysfunction and memory deficits in the alcoholic Korsakoff syndrome and Alzheimer-type dementia. *Brain, 114*(1 A), 117-137, doi:https://doi.org/10.1093/oxfordjournals.brain.a101852.

Leng, N. R. C., & Parkin, A. J. (1988). Double dissociation of frontal dysfunction in organic amnesia. *British Journal of Clinical Psychology, 27*(4), 359-362, doi:10.1111/j.2044-8260.1988.tb00800.x.

Maharasingam, M., Macniven, A. B., & Mason, J. (2013). Executive functioning in chronic alcoholism and Korsakoff syndrome. *Journal Of Clinical And Experimental Neuropsychology, 35*(5), 501-508. doi:http://dx.doi.org/10.1080/13803395.2013.795527

Mazzucchi, A., Capitani, E., Poletti, A., Posteraro, L., Bocelli, G., Campari, F., & Parma, M. (1987). Discriminant analysis of WAIS results in different types of dementia and depressed patients. *Functional Neurology, 2*(2), 155-163.

O'Carroll, R. E., Moffoot, A., Ebmeier, K. P., & Goodwin, G. M. (1992). Estimating pre-morbid intellectual ability in the Alcoholic Korsakoff Syndrome. *Psychological Medicine, 22*(4), 903-909. doi:http://dx.doi.org/10.1017/S0033291700038472

Oscar-Berman, M., Clancy, J. P., & Weber, D. A. (1993). Discrepancies between IQ and memory scores in alcoholism and aging. *Clinical Neuropsychologist, 7*(3), 281-296. doi:http://dx.doi.org/10.1080/13854049308401899

Oscar-Berman, M., Kirkley, S. M., Gansler, D. A., & Couture, A. (2004). Comparisons of Korsakoff and non-Korsakoff alcoholics on neuropsychological tests of prefrontal brain functioning. *Alcoholism: Clinical and Experimental Research, 28*(4), 667-675. doi:http://dx.doi.org/10.1097/01.ALC.0000122761.09179.B9

Oudman, E., Postma, A., Van der Stigchel, S., Appelhof, B., Wijnia, J. W., & Nijboer, T. C. (2014). The Montreal Cognitive Assessment (MoCA) is superior to the Mini Mental State Examination (MMSE) in detection of Korsakoff's syndrome. *The Clinical Neuropsychologist, 28*(7), 1123-1132, doi:10.1080/13854046.2014.960005.

Piekema, C., Fernández, G., Postma, A., Hendriks, M. P. H., Wester, A. J., & Kessels, R. P. C. (2007). Spatial and non-spatial contextual working memory in patients with diencephalic or hippocampal dysfunction. *Brain Research, 1172*, 103-109. doi:http://dx.doi.org/10.1016/j.brainres.2007.07.066

Pitel, A. L., Beaunieux, H., Witkowski, T., Vabret, F., de la Sayette, V., Viader, F., . . . Eustache, F. (2008). Episodic and working memory deficits in alcoholic Korsakoff patients: The continuity theory revisited. *Alcoholism: Clinical and Experimental Research, 32*(7), 1229-1241. doi:http://dx.doi.org/10.1111/j.1530-0277.2008.00677.x

Rensen, Y. C. M., Kessels, R. P. C., Migo, E. M., Wester, A. J., Eling, P. A. T. M., & Kopelman, M. D. (2016). Personal semantic and episodic autobiographical memories in Korsakoff syndrome: A comparison of interview methods. *Journal Of Clinical And Experimental Neuropsychology, 39*(6), 534-546. doi:10.1080/13803395.2016.1248811

Rensen, Y. C. M., Oosterman, J. M., van Damme, J. E., Griekspoor, S. I. A., Wester, A. J., Kopelman, M. D., & Kessels, R. P. C. (2015). Assessment of Confabulation in Patients with Alcohol-Related Cognitive Disorders: The Nijmegen-Venray Confabulation List (NVCL-20). *The Clinical Neuropsychologist, 29*(6), 804-823. doi:10.1080/13854046.2015.1084377

Rensen, Y. C. M., Oosterman, J. M., Walvoort, S. J. W., Eling, P. A. T. M., & Kessels, R. P. C. (2017). Intrusions and provoked and spontaneous confabulations on memory tests in Korsakoff’s syndrome. *Journal Of Clinical And Experimental Neuropsychology, 39*(2), 101-111. doi:10.1080/13803395.2016.1204991

Shoqeirat, M. A., Mayes, A., MacDonald, C., Meudell, P., & Pickering, A. (1990). Performance on tests sensitive to frontal lobe lesions by patients with organic amnesia: Leng & Parkin revisited. *The British Journal of Clinical Psychology, 29*, 401-408, doi:10.1111/j.2044-8260.1990.tb00903.x.

Taylor, M. J., & Heaton, R. K. (2001). Sensitivity and specificity of WAIS-III/WMS-III demographically corrected factor scores in neuropsychological assessment. *Journal of the International Neuropsychological Society: JINS, 7*(7), 867-874.

Taylor, R., & O'Carroll, R. (1995). Cognitive estimation in neurological disorders. *British Journal of Clinical Psychology, 34*(2), 223-228, doi:http://dx.doi.org/10.1111/j.2044-8260.1995.tb01456.x.

Van Den Berg, E., Nys, G. M. S., Brands, A. M. A., Ruis, C., Van Zandvoort, M. J. E., & Kessels, R. P. C. (2009). The Brixton Spatial Anticipation Test as a test for executive function: Validity in patient groups and norms for older adults. *Journal of the International Neuropsychological Society : JINS, 15*(5), 695-703. doi:http://dx.doi.org/10.1017/S1355617709990269

van Oort, R., & Kessels, R. P. C. (2009). Executive dysfunction in Korsakoff's syndrome: Time to revise the DSM criteria for alcohol-induced persisting amnestic disorder? *International Journal of Psychiatry in Clinical Practice, 13*(1), 78-81. doi:http://dx.doi.org/10.1080/13651500802308290

Weintraub, S., Peavy, G. M., O'Connor, M., Johnson, N. A., Acar, D., Sweeney, J., et al. (2000). Three Words - Three Shapes: A clinical test of memory. *Journal Of Clinical And Experimental Neuropsychology, 22*(2), 267-278, doi:10.1076/1380-3395(200004)22:2;1-1;FT267.

Welch, L. W., Nimmerrichter, A., Gilliland, R., King, D. E., & Martin, P. R. (1997). “Wineglass” Confabulations Among Brain- Damaged Alcoholics on the Wechsler Memory Scale-Revised Visual Reproduction Subtest*. *Cortex, 33*(3), 543-551. doi:http://dx.doi.org/10.1016/S0010-9452(08)70235-1

Wester, A. (2007). De Rivermead Behavioural Memory Test: Een maat voor het alledaagse geheugen van Korsakovpatiënten. [The Rivermead Behavioural Memory Test: a measure of everyday memory of Korsakoff patients]. *Tijdschrift voor Neuropsychologie, 1*, 30-41.

Wester, A. J., Leenders, P., Egger, J. I., & Kessels, R. P. C. (2013). Ceiling and floor effects on the Rivermead Behavioural Memory Test in patients with alcohol-related memory disorders and healthy participants. *International Journal of Psychiatry in Clinical Practice, 17*(4), 286-291. doi:http://dx.doi.org/10.3109/13651501.2013.813553

Wester, A. J., Roelofs, R. L., Egger, J. I., & Kessels, R. P. C. (2014). Assessment of alcohol-related memory deficits: A comparison between the Rivermead Behavioural Memory Test and the California Verbal Learning Test. *Brain Impairment, 15*(1), 18-27. doi:http://dx.doi.org/10.1017/BrImp.2014.6

Wester, A. J., van Herten, J. C., Egger, J. I., & Kessels, R. P. C. (2013). Applicability of the Rivermead Behavioural Memory Test - Third Edition (RBMT-3) in Korsakoff's syndrome and chronic alcoholics. *Neuropsychiatric Disease And Treatment, 9*, 875-881. doi:10.2147/NDT.S44973

Wester, A. J., Westhoff, J., Kessels, R. P. C., & Egger, J. I. M. (2013). The Montreal Cognitive Asesment (MoCA) as a measure of severity of amnesia in patients with alcohol-related cognitive impairments and Korsakof syndrome. *Clinical Neuropsychiatry, 10*(3-4), 134-141.

Woodburn, K., & Johnstone, E. (1999a). Early-onset dementia in Lothian, Scotland: an analysis of clinical features and patterns of decline. *Health bulletin, 57*(6), 384-392.

Woodburn, K., & Johnstone, E. (1999b). I. Measuring the decline of a population of people with early-onset dementia in Lothian, Scotland. *International Journal Of Geriatric Psychiatry, 14*(5), 355-361. doi:http://dx.doi.org/10.1002/(SICI)1099-1166(199905)14:5<355::AID-GPS913>3.0.CO;2-P

**References (62 studies extracted only in phase one)**

Alderdice, F. A., McGuinness, C., & Brown, K. (1994). Identification of subtypes of problem drinkers based on neuropsychological performance. *The British Journal of Clinical Psychology, 33* 483-498. doi:10.1111/j.2044-8260.1994.tb01145.x

Asada, T., Takaya, S., Takayama, Y., Yamauchi, H., Hashikawa, K., & Fukuyama, H. (2010). Reversible alcohol-related dementia: a five-year follow-up study using FDG-PET and neuropsychological tests. *Internal Medicine, 49*(4), 283-287. doi:10.2169/internalmedicine.49.2662

Beaunieux, H., Pitel, A. L., Witkowski, T., Vabret, F., Viader, F., & Eustache, F. (2013). Dynamics of the cognitive procedural learning in alcoholics with Korsakoff's syndrome. *Alcoholism, Clinical And Experimental Research, 37*(6), 1025-1032. doi:10.1111/acer.12054

Besson, J. A., Crawford, J. R., Parker, D. M., & Smith, F. W. (1989). Magnetic resonance imaging in Alzheimer's disease, multi-infarct dementia, alcoholic dementia and Korsakoff's psychosis. *Acta Psychiatrica Scandinavica, 80*(5), 451-458. doi:10.1111/j.1600-0447.1989.tb03005.x

Bigler, E. D., Nelson, J. E., & Schmidt, R. D. (1989). Mamillary body atrophy identified by magnetic resonance imaging in alcohol amnestic (Korsakoff's) syndrome: Neuropsychological correlates. *Neuropsychiatry, Neuropsychology and Behavioral Neurology, 2*(3), 189-201.

Blansjaar, B. A., Takens, H., & Zwinderman, A. H. (1992). The course of alcohol amnestic disorder: a three-year follow-up study of clinical signs and social disabilities. *Acta Psychiatrica Scandinavica, 86*(3), 240-246. doi:10.1111/j.1600-0447.1992.tb03260.x

Borsutzky, S., Fujiwara, E., Brand, M., & Markowitsch, H. J. (2008). Confabulations in alcoholic Korsakoff patients. *Neuropsychologia, 46*(13), 3133-3143. doi:http://dx.doi.org/10.1016/j.neuropsychologia.2008.07.005

Brand, M., Fujiwara, E., Borsutzky, S., Kalbe, E., Kessler, J., & Markowitsch, H. J. (2005). Decision-making deficits of korsakoff patients in a new gambling task with explicit rules: associations with executive functions. *Neuropsychology, 19*(3), 267-277.

Brand, M., Fujiwara, E., Kalbe, E., Steingass, H. P., Kessler, J., & Markowitsch, H. J. (2003). Cognitive estimation and affective judgments in alcoholic Korsakoff patients. *Journal Of Clinical And Experimental Neuropsychology, 25*(3), 324-334.

Brand, M., Kalbe, E., Fujiwara, E., Huber, M., & Markowitsch, H. J. (2003). Cognitive estimation in patients with probable Alzheimer’s disease and alcoholic Korsakoff patients. *Neuropsychologia, 41*(5), 575-584. doi:http://dx.doi.org/10.1016/S0028-3932(02)00183-5

Butters, N., Granholm, E., Salmon, D. P., Grant, I., & Wolfe, J. (1987). Episodic and semantic memory: a comparison of amnesic and demented patients. *Journal Of Clinical And Experimental Neuropsychology, 9*(5), 479-497. doi:http://dx.doi.org/10.1080/01688638708410764

Canaris, C. A., & Jurd, S. (1991). The diagnosis of alcohol-related brain damage: a retrospective study in alcoholics undergoing in-patient rehabilitation. *Drug And Alcohol Review, 10*(1), 85-88.

Carlen, P. L., McAndrews, M. P., Weiss, R. T., Dongier, M., Hill, J.-M., Menzano, E., . . . Eastwood, M. R. (1994). Alcohol-related dementia in the institutionalized elderly. *Alcoholism: Clinical and Experimental Research, 18*(6), 1330-1334. doi:http://dx.doi.org/10.1111/j.1530-0277.1994.tb01432.x

Charter, R. A., Walden, D. K., & Hoffman, C. (1998). Interscorer reliabilities for memory and localization scores of the tactual performance test. *Clinical Neuropsychologist, 12*(2), 245-247.

Dirksen, C. L., Howard, J. A., Cronin-Golomb, A., & Oscar-Berman, M. (2006). Patterns of prefrontal dysfunction in alcoholics with and without Korsakoff's syndrome, patients with Parkinson's disease, and patients with rupture and repair of the anterior communicating artery. *Neuropsychiatric Disease And Treatment, 2*(3), 327-339. doi:http://dx.doi.org/10.2147/nedt.2006.2.3.327

Emsley, R., Smith, R., Roberts, M., & Kapnias, S. (1996). Magnetic resonance imaging in alcoholic Korsakoff's syndrome: Evidence for an association with alcoholic dementia. *Alcohol and Alcoholism, 31*(5), 479-486. doi:http://dx.doi.org/10.1093/oxfordjournals.alcalc.a008182

Everall, I. P. (1988). Language disorder: A presenting symptom of alcohol dementia. *British Journal Of Addiction, 83*(4), 433-436. doi:http://dx.doi.org/10.1111/j.1360-0443.1988.tb00491.x

Fama, R., Marsh, L., & Sullivan, E. V. (2004). Dissociation of remote and anterograde memory impairment and neural correlates in alcoholic Korsakoff syndrome. *Journal of the International Neuropsychological Society, 10*(3), 427-441. doi:http://dx.doi.org/10.1017/S135561770410310X

Fujiwara, E., Brand, M., Borsutzky, S., Steingass, H. P., & Markowitsch, H. J. (2008). Cognitive performance of detoxified alcoholic Korsakoff syndrome patients remains stable over two years. *Journal Of Clinical And Experimental Neuropsychology, 30*(5), 576-587. doi:http://dx.doi.org/10.1080/13803390701557271

Gilchrist, G., & Morrison, D. S. (2005). Prevalence of alcohol related brain damage among homeless hostel dwellers in Glasgow. *European Journal of Public Health, 15*(6), 587-588. doi:https://doi.org/10.1093/eurpub/cki036

Haxby, J. V., Lundgren, S. L., & Morley, G. K. (1983). Short-term retention of verbal, visual shape and visuospatial location information in normal and amnesic subjects. *Neuropsychologia, 21*(1), 25-33.

Heinrichs, R. W. (1994). Performance on tests of diencephalic-hippocampal verbal memory function in schizophrenia, Korsakoff's syndrome and personality disorder. *Schizophrenia Research, 13*(2), 127-132. doi:http://dx.doi.org/10.1016/0920-9964(94)90093-0

Hildebrandt, H., Brokate, B., Eling, P., & Lanz, M. (2004). Response Shifting and Inhibition, but Not Working Memory, Are Impaired After Long-Term Heavy Alcohol Consumption. *Neuropsychology, 18*(2), 203-211. doi:http://dx.doi.org/10.1037/0894-4105.18.2.203

Irle, E., Kaiser, P., & Naumann-Stoll, G. (1990). Differential patterns of memory loss in patients with Alzheimer's disease and Korsakoff's disease. *International Journal of Neuroscience, 52*(1-2), 67-77. doi:http://dx.doi.org/10.3109/00207459008994245

Jacobson, R. R. (1989). Alcoholism, Korsakoff's syndrome and the frontal lobes. *Behavioural Neurology, 2*(1), 25-38. doi:http://dx.doi.org/10.1155/1989/847937

Jacobson, R. R., Acker, C. F., & Lishman, W. A. (1990). Patterns of neuropsychological deficit in alcoholic Korsakoff's syndrome. *Psychological Medicine, 20*(2), 321-334. doi:http://dx.doi.org/10.1017/S0033291700017633

Jacobson, R. R., & Lishman, W. A. (1987). Selective memory loss and global intellectual deficits in alcoholic Korsakoff's syndrome. *Psychological Medicine, 17*(3), 649-655. doi:http://dx.doi.org/10.1017/S0033291700025885

Joyce, E. M., & Robbins, T. W. (1991). Frontal lobe function in Korsakoff and non-Korsakoff alcoholics: Planning and spatial working memory. *Neuropsychologia, 29*(8), 709-723. doi:http://dx.doi.org/10.1016/0028-3932(91)90067-I

Kessels, R. P. C., Kortrijk, H. E., Wester, A. J., & Nys, G. M. S. (2008). Confabulation behavior and false memories in Korsakoff's syndrome: Role of source memory and executive functioning. *Psychiatry and Clinical Neurosciences, 62*(2), 220-225. doi:http://dx.doi.org/10.1111/j.1440-1819.2008.01758.x

Kessler, J., Markowitsch, H. J., & Bast-Kessler, C. (1987). Memory of alcoholic patients, including Korsakoff's, tested with a Brown-Peterson paradigm. *Archiv für Psychologie, 139*(2), 115-132.

Kixmiller, J. S., Verfaellie, M., Mather, M. M., & Cermak, L. S. (2000). Role of perceptual and organizational factors in amnesics' recall of the Rey-Osterrieth Complex Figure: A comparison of three amnesic groups. *Journal Of Clinical And Experimental Neuropsychology, 22*(2), 198-207. doi:http://dx.doi.org/10.1076/1380-3395(200004)22:2;1-1;FT198

Kopelman, M. D. (1985). Rates of forgetting in Alzheimer-type dementia and Korsakoff's syndrome. *Neuropsychologia, 23*(5), 623-638. doi:http://dx.doi.org/10.1016/0028-3932(85)90064-8

Kopelman, M. D. (1989). Remote and autobiographical memory, temporal context memory and frontal atrophy in Korsakoff and Alzheimer patients. *Neuropsychologia, 27*(4), 437-460. doi:http://dx.doi.org/10.1016/0028-3932(89)90050-X

Kopelman, M. D. (1991). Non-verbal, short-term forgetting in the alcoholic Korsakoff syndrome and Alzheimer-type dementia. *Neuropsychologia, 29*(8), 737-747. doi:http://dx.doi.org/10.1016/0028-3932(91)90069-K

Krabbendam, L., Visser, P. J., Derix, M. M. A., Verhey, F., Hofman, P., Verhoeven, W., . . . Jolles, J. (2000). Normal cognitive performance in patients with chronic alcoholism in contrast to patients with Korsakoff's syndrome. *The Journal Of Neuropsychiatry And Clinical Neurosciences, 12*(1), 44-50. doi:10.1176/jnp.12.1.44

Labudda, K., Todorovski, S., Markowitsch, H. J., & Brand, M. (2008). Judgment and memory performance for emotional stimuli in patients with alcoholic Korsakoff syndrome. *Journal Of Clinical And Experimental Neuropsychology, 30*(2), 1-12. doi:http://dx.doi.org/10.1080/13803390701363811

Leng, N. R. C., & Parkin, A. J. (1988). Double dissociation of frontal dysfunction in organic amnesia. *British Journal of Clinical Psychology, 27*(4), 359-362. doi:10.1111/j.2044-8260.1988.tb00800.x

Longmore, B. E., & Knight, R. G. (1988). The effect of intellectual deterioration on retention deficits in amnesic alcoholics. *J Abnorm Psychol, 97*(4), 448-454. doi:http://dx.doi.org/10.1037/0021-843X.97.4.448

Mair, R. G., McEntee, W. J., & Zatorre, R. J. (1985). Monoamine activity correlates with psychometric deficits in Korsakoff's disease. *Behavioural Brain Research, 15*(3), 247-254. doi:http://dx.doi.org/10.1016/0166-4328(85)90179-2

Malerstein, A. J., & Belden, E. (1968). WAIS, SILS, and PPVT in Korsakoff's syndrome. *Archives Of General Psychiatry, 19*(6), 743-750.

Mayes, A. R., Downes, J. J., Symons, V., & Shoqeirat, M. (1994). Do amnesics forget faces pathologically fast? *Cortex: A Journal Devoted to the Study of the Nervous System and Behavior, 30*(4), 543-563. doi:https://doi.org/10.1016/S0010-9452(13)80235-3

Mimura, M., Kinsbourne, M., & O'Connor, M. (2000). Time estimation by patients with frontal lesions and by Korsakoff amnesics. *Journal of the International Neuropsychological Society, 6*(5), 517-528. doi:http://dx.doi.org/10.1017/S1355617700655017

Munro, C. A., Saxton, J., & Butters, M. A. (2001). Alcohol dementia: “cortical” or “subcortical” dementia? *Archives of Clinical Neuropsychology, 16*(6), 523-533. doi:http://dx.doi.org/10.1016/S0887-6177(00)00063-9

Neville, H. J., & Folstein, M. F. (1979). Performance on three cognitive tasks by patients with dementia, depression or Korsakov's syndrome. *Gerontology, 25*(5), 285-290. doi:10.1159/000212353

Oosterman, J. M., de Goede, M., Wester, A. J., van Zandvoort, M. J. E., & Kessels, R. P. C. (2011). Perspective taking in Korsakoff's syndrome: The role of executive functioning and task complexity. *Acta Neuropsychiatrica, 23*(6), 302-308. doi:http://dx.doi.org/10.1111/j.1601-5215.2011.00552.x

Oslin, D. W., & Cary, M. S. (2003). Alcohol-related dementia: Validation of diagnostic criteria. *The American Journal of Geriatric Psychiatry, 11*(4), 441-447. doi:http://dx.doi.org/10.1176/appi.ajgp.11.4.441

Osuntokun, B. O., Hendrie, H. C., Fisher, K., McMahon, D., & Brittain, H. (1994). The diagnosis of dementia associated with alcoholism: a preliminary report of a new approach. *West African journal of medicine, 13*(3), 160-163.

Pitel, A. L., Beaunieux, H., Guillery-Girard, B., Witkowski, T., de la Sayette, V., Viader, F., . . . Eustache, F. (2009). How do Korsakoff patients learn new concepts? *Neuropsychologia, 47*(3), 879-886. doi:http://dx.doi.org/10.1016/j.neuropsychologia.2008.12.019

Rubin, D. C., Olson, E. H., Richter, M., & Butters, N. (1981). Memory for prose in Korsakoff and schizophrenic populations. *International Journal of Neuroscience, 13*(2-3), 81-85. doi:10.3109/00207458109043304

Ryan, C., & Butters, N. (1980). Further evidence for a continuum-of-impairment encompassing male alcoholic Korsakoff patients and chronic alcoholic men. *Alcoholism, Clinical And Experimental Research, 4*(2), 190-198. doi:10.1111/j.1530-0277.1980.tb05634.x

Saxton, J., Munro, C. A., Butters, M. A., Schramke, C., & McNeil, M. A. (2000). Alcohol, dementia, and Alzheimer's disease: Comparison of neuropsychological profiles. *Journal of Geriatric Psychiatry and Neurology, 13*(3), 141-149. doi:10.1177/089198870001300308

Schmidt, K. S., Gallo, J. L., Ferri, C., Giovannetti, T., Sestito, N., Libon, D. J., & Schmidt, P. S. (2005). The neuropsychological profile of alcohol-related dementia suggests cortical and subcortical pathology. *Dementia And Geriatric Cognitive Disorders, 20*(5), 286-291. doi:10.1159/000088306

Shimamura, A. P., Jernigan, T. L., & Squire, L. R. (1988). Korsakoff's syndrome: Radiological (CT) findings and neuropsychological correlates. *Journal of Neuroscience, 8*(11), 4400-4410.

Shimamura, A. P., Salmon, D. P., Squire, L. R., & Butters, N. (1987). Memory dysfunction and word priming in dementia and amnesia. *Behavioral Neuroscience, 101*(3), 347-351. doi:http://dx.doi.org/10.1037/0735-7044.101.3.347

Shoqeirat, M. A., & Mayes, A. R. (1991). Disproportionate incidental spatial-memory and recall deficits in amnesia. *Neuropsychologia, 29*(8), 749-769. doi:https://doi.org/10.1016/0028-3932(91)90070-O

Spiegel, D. R., & Jim, K. J. (2011). A case of probable korsakoff's syndrome: A syndrome of frontal lobe and diencephalic structural pathogenesis and a comparison with medial temporal lobe dementias. *Innovations in Clinical Neuroscience, 8*(6), 15-19.

Squire, L. R., & Shimamura, A. P. (1986). Characterizing amnesic patients for neurobehavioral study. *Behavioral Neuroscience, 100*(6), 866-877. doi:http://dx.doi.org/10.1037/0735-7044.100.6.866

Sturmey, P., Gatherer, A., Ghadiali, E., Hallett, S., & Searle, Y. (1993). The Wechsler Adult Intelligence Scale—Revised (WAIS-R): factor structure in a British, neurologically impaired population. *Personality and Individual Differences, 14*(1), 255-257. doi:http://dx.doi.org/10.1016/0191-8869(93)90198-C

Tuck, R. R., & Jackson, M. (1991). Social, neurological and cognitive disorders in alcoholics. *Medical Journal of Australia, 155*(4), 225-229.

van Geldorp, B., Bergmann, H. C., Robertson, J., Wester, A. J., & Kessels, R. P. C. (2012). The interaction of working memory performance and episodic memory formation in patients with Korsakoff's amnesia. *Brain Research, 1433*, 98-103. doi:http://dx.doi.org/10.1016/j.brainres.2011.11.036

Visser, P. J., Krabbendam, L., Verhey, F. R. J., Hofman, P. A. M., Verhoeven, W. M. A., Tuinier, S., . . . Jolles, J. (1999). Brain correlates of memory dysfunction in alcoholic Korsakoff's syndrome. *Journal of Neurology, Neurosurgery & Psychiatry, 67*(6), 774-778. doi:http://dx.doi.org/10.1136/jnnp.67.6.774

Zinn, S., Bosworth, H. B., Edwards, C. L., Logue, P. E., & Swartzwelder, H. S. (2003). Performance of recently detoxified patients with alcoholism on a neuropsychological screening test.
